# An Integrated “Multi-Omics” Comparison of Embryo and Endosperm Tissue-Specific Features and Their Impact on Rice Seed Quality

**DOI:** 10.3389/fpls.2017.01984

**Published:** 2017-11-22

**Authors:** Marc Galland, Dongli He, Imen Lounifi, Erwann Arc, Gilles Clément, Sandrine Balzergue, Stéphanie Huguet, Gwendal Cueff, Béatrice Godin, Boris Collet, Fabienne Granier, Halima Morin, Joseph Tran, Benoit Valot, Loïc Rajjou

**Affiliations:** ^1^IJPB, Institut Jean-Pierre Bourgin (INRA, AgroParisTech, CNRS, Université Paris-Saclay), Saclay Plant Sciences (SPS), Versailles, France; ^2^Wuhan Botanical Garden, Chinese Academy of Sciences, Wuhan, China; ^3^IPS2, Institute of Plant Sciences Paris-Saclay (INRA, CNRS, Université Paris-Sud, Université d'Evry, Université Paris-Diderot, Sorbonne Paris-Cité, Université Paris-Saclay), POPS-Transcriptomic Platform, Saclay Plant Sciences (SPS), Orsay, France; ^4^GQE-Le Moulon, Génétique Quantitative et Evolution (INRA Université Paris-Sud, CNRS, AgroParisTech, Université Paris-Saclay), PAPPSO-Plateforme d'Analyse Protéomique de Paris Sud-Ouest, Saclay Plant Sciences (SPS), Gif-sur-Yvette, France

**Keywords:** rice, seed, endosperm, embryo, multi-omics, translation, starch, glutelins

## Abstract

Although rice is a key crop species, few studies have addressed both rice seed physiological and nutritional quality, especially at the tissue level. In this study, an exhaustive “multi-omics” dataset on the mature rice seed was obtained by combining transcriptomics, label-free shotgun proteomics and metabolomics from embryo and endosperm, independently. These high-throughput analyses provide a new insight on the tissue-specificity related to rice seed quality. Foremost, we pinpointed that extensive post-transcriptional regulations occur at the end of rice seed development such that the embryo proteome becomes much more diversified than the endosperm proteome. Secondly, we observed that survival in the dry state in each seed compartment depends on contrasted metabolic and enzymatic apparatus in the embryo and the endosperm, respectively. Thirdly, it was remarkable to identify two different sets of starch biosynthesis enzymes as well as seed storage proteins (glutelins) in both embryo and endosperm consistently with the supernumerary embryo hypothesis origin of the endosperm. The presence of a putative new glutelin with a possible embryonic favored abundance is described here for the first time. Finally, we quantified the rate of mRNA translation into proteins. Consistently, the embryonic panel of protein translation initiation factors is much more diverse than that of the endosperm. This work emphasizes the value of tissue-specificity-centered “multi-omics” study in the seed to highlight new features even from well-characterized pathways. It paves the way for future studies of critical genetic determinants of rice seed physiological and nutritional quality.

## Introduction

Seeds of high nutritional and physiological quality are essential for the benefit of mankind. Seeds are also at the forefront for the preservation of biodiversity through the plant conservation strategies in seed banks (Wyse Jackson and Kennedy, 2009; Westengen et al., 2013; Hay and Whitehouse, 2017). The quality of seeds comprises physiological, ecological and nutritional traits for agriculture, agroecology, and agro-food system. In terms of botanical provenance, the mature seed of Angiosperms is a patchwork of maternal and filial tissues (Walbot and Evans, 2003; Olsen, 2004; Nowack et al., 2010; Lafon-Placette and Kohler, 2014). The double fertilization of the haploid egg cell and diploid central cell by the two haploid sperm cells gives rise to a diploid embryo and to a triploid endosperm respectively. In addition, the seed coat (testa) is composed of several cell layers coming from the mother plant ovule and ovary. In cereals (*Poaceae*), the embryo is composed of the embryonic axis surrounded by a single cotyledon (scutellum) and it will form the future seedling. The mature endosperm is composed of four differentiated regions: the endosperm transfer cell (ETC) region, the embryo-surrounding region (ESR), the aleurone layer (AL), and the starchy endosperm (SE) cells (Olsen, 2004). While the ETC and AL cells remain alive at the end of cereal seed development, most of the ESR and SE cells have undergone programmed cell death (PCD) with characteristic DNA laddering and organelle degradation.

From an evolutionary point of view, one hypothesis stipulates that the endosperm would have derived from a supernumerary embryo originating from double fertilization and that would have evolved into an embryo-supporting structure (Friedman, 1998). Subsequently, the endosperm has evolved multiple roles related to the embryo. First, the endosperm protects the embryo from atmospheric oxygen that eventually leads to the formation of hydroperoxides and cell death (De Giorgi et al., 2015). In addition, critical cross-talk between abscisic acid (ABA) and gibberellin (GA) regulating seed development, size, dormancy or storage breakdown during germination are also the results of endosperm—embryo interactions (Fincher, 1989; Penfield et al., 2006; Bethke et al., 2007; Folsom et al., 2014; Yan et al., 2014; Bassel, 2016). Still, so far, few reports have addressed seed quality issues in terms of functional tissue specialization.

During seed maturation, orthodox seeds acquire desiccation tolerance (DT) and storability (longevity) as defined by the ability to withstand extreme water loss (to values around 0.1 g water per gram of dry weight) and to survive in the dry state (Hoekstra et al., 2001; Alpert, 2005). Among the mechanisms that promote DT, the formation of a glassy cytoplasm and the subsequent decrease in molecular mobility is positively correlated with seed longevity (Buitink and Leprince, 2008). During the late phase of seed maturation, the accumulation of late embryogenesis abundant (LEA) proteins, heat shock proteins (HSPs), antioxidants and non-reducing sugars all together contribute to glassy state establishment (Boudet et al., 2006; Farrant and Moore, 2011; Kaur et al., 2016; Sano et al., 2016). How different tissues cooperate to establish seed DT and storability is still unclear especially in cereals with a persistent endosperm. Among the few existing studies, a proteomic analysis on maize *viviparous5* (*vp5*) mutant showed that LEA and HSPs were affected in the ABA-deficient embryos of the *vp5* mutants contrarily to the endosperm (Wu et al., 2014). Furthermore, a consequence of lipid degradation during storage, detoxification of the lipid peroxidation by-product malondialdehyde (MDA) by the rice aldehyde dehydrogenase 7 (OsALDH7) proved to be essential for DT (Shin et al., 2009). The null rice mutants of OsALDH7 showed increased MDA resulting in reduced seed viability (Shin et al., 2009). Yet, the exact site of MDA generation during dry storage and seed aging remains unknown. The impact of active lipid degradation on rice seed quality was further reinforced by transgenic analysis of two rice lipoxygenases OsLOX2 and OsLOX3 since silencing and overexpression of these two enzymes acts in opposite directions on seed germination and longevity (Huang et al., 2014; Xu et al., 2015). Interestingly, the suppression of *OsLOX3* expression in the rice endosperm improved resistance to seed aging (Xu et al., 2015). The endosperm outer cuticle layer permeability can also preserve the embryonic components from oxidation by atmospheric oxygen (De Giorgi et al., 2015). A recent paper showed that during rice seed aging, the endosperm and embryo were differentially affected by seed aging in particular regarding glycolytic enzymes that decreased in abundance in the endosperm while increasing in the embryo (Zhang et al., 2016). Last but not least, seed storage proteins can also buffer the oxidative stress caused by seed aging as shown for Arabidopsis cruciferins (Rajjou et al., 2008; Nguyen et al., 2015). Despite these relevant studies, the majority of the molecular determinants of both DT and seed longevity in different seed tissues are still to be established especially in the embryo of cereal crops.

The generation of energy, digestion of seed storage proteins (SSPs), carbohydrates and/or triacylglycerols (TAGs) proved to be of paramount importance to obtain highly vigorous seeds. Within a few hours of imbibition, the seed embryo is rapidly resuming respiration thanks to the presence of a functional electron transport chain in undifferentiated pro-mitochondria (Ehrenshaft and Brambl, 1990; Logan et al., 2001; Howell et al., 2006). Later on, the differentiation of these pro-mitochondria in fully functional mitochondria participates to full metabolic resumption through establishment of the tricarboxylic acid cycle (TCA) (Lawlor and Vince, 2014). In contrast, it is less clear how the AL cells produce the requested energy to synthesize the large amounts of α-amylases for starch degradation (Fincher, 1989). During barley seed development, the inner SE is mildly to severely hypoxic while the AL is not (Rolletschek et al., 2011). Still, the precise investigation of oxygen requirement and consumption during germination in the different cereal seed tissues remains to be established.

The metabolic system for mRNA translation and protein synthesis is the most energy-requiring process in most organisms. Through the use of the translational inhibitor cycloheximide, it has been shown that translation of stored mRNAs is necessary and sufficient for both Arabidopsis and rice germination (Rajjou et al., 2004; Sano et al., 2012, 2013). On the other hand, DNA transcription by RNA polymerase II is necessary for seed vigor and seedling growth (Rajjou et al., 2004; Sano et al., 2012). In cereal seeds, the importance of mRNA translation in the AL during germination is crucial. Indeed, a strong synthesis and accumulation of α-amylases, in response to gibberellic acid, participates to starch mobilization that in turns fuel the germinating embryo with oligosaccharides (Fincher, 1989). Interestingly, the scutellum also provides α-amylases contributing to the amylolytic activity of the starch endosperm during germination (Subbarao et al., 1998) suggesting a close relationship between endosperm tissues and embryo. Nevertheless, only few studies have addressed the precise content of the cereal endosperm in terms of translational machinery especially compared to that of the cereal embryo that was historically used as a model cell-free system to translate mRNAs *in vitro* (Takai and Endo, 2010).

As a model species for cereals with a well-annotated genome (International Rice Genome Sequencing Project, 2005), rice seeds are widely studied by taking advantage of “omics” approaches (Koller et al., 2002; Tarpley et al., 2005; Xu et al., 2008; Jiao et al., 2009; Wang et al., 2010; Lee and Koh, 2011; Nguyen et al., 2012; Xue et al., 2012). Most studies on cereal seed focused either on the isolated embryo (Howell et al., 2009; Kim et al., 2009; Han et al., 2014) or on the whole seed (Yang et al., 2007). In contrast, a small number of works were performed on both the embryo and endosperm in the same experiment (Gallardo et al., 2007; Sreenivasulu et al., 2008).

Here, we used an integrated “multi-omics” approach combining transcriptomics, label-free quantitative shotgun proteomics and gas chromatography coupled to mass spectrometry (GC-MS)-based metabolomics on dry mature rice seeds from a reference rice cultivar (*Oryza sativa* ssp *japonica* cv Nipponbare). The present work was firstly aimed at comparing the compartmentalization of nutritionally relevant pathways between the endosperm and embryo (nutritional quality). These pathways could be further improved through metabolic engineering based on knowledge of the fine composition of both embryo and endosperm. Secondly, we highlighted genes potentially important for seed storability and germination (agricultural quality). Thirdly, a targeted study of the factors associated with the seed storage compounds (starch and proteins) were analyzed carefully. Altogether, these exhaustive datasets emphasize determinants of rice seed quality in a tissue-specific manner.

## Methods

### Rice biological material

Dry mature rice seeds (*Oryza sativa* ssp *japonica* cv Nipponbare) were harvested in September 2011 at the “Centre Français du Riz” (Mas du Sonnailler, Arles, France). At the lab, seeds were dehulled and dissected in one embryo (E0) and one endosperm (A0) fraction with a sharp scalpel. The white rice (SE) fraction was obtained thanks to a lab bench rice milling machine as previously described (Galland et al., 2014a). Dry weight was determined on 10 bulks comprising 10 rice seeds. The corresponding E0, A0, and SE were placed in a dry oven at 105°C for 48 h and weighted on a precision lab balance (XP204, Mettler-Toledo, France).

### Optical and confocal microscopy

Fixation, inclusion into historesin and cutting of 5 μm semi-thin sections were done exactly as previously described (Galland et al., 2014a). Proteins and complex carbohydrates were revealed using a Periodic Acid (PA)—Schiff /Naphthol Blue Black (NBB) staining. Samples semi-thin sections (5 μm) were first hydrolyzed 5 min in 1% periodic acid (w/v), rinsed with tap water and distilled water and then colored with Schiff's reagent for 10 min in complete darkness. Subsequently, sample slices were washed with sulfurous water that contains 5% (w/v) sodium metabisulfite, 250 mM HCl and distilled water for 1 min before washing with tap water and distilled water. NBB staining was done using a pre-heated (65°C) working solution that contains 0.1% (w/v) NBB, 10% (v/v) acetic acid and distilled water in which samples were placed for 5 min. One final thorough washing was done in tap water and samples sections were finally placed in acetic acid (7%, v/v) for 1 min or more if PA-Schiff staining was too weak. Finally, samples sections were mounted between glass slides in glycerol (Histomount, National Diagnostics, UK) for visualization before imaging with Leica optical microscopy (Leica Zeiss Axioplan, Leica Microsystems, Wetzlar, Germany).

Neutral lipids were imaged by confocal scanning fluorescence microscopy (Leica TCS SP2, Leica Microsystèmes SAS, France) using the Nile red dye (Greenspan et al., 1985). For neutral lipid observation, 100 μm wide sections of rice dry mature seeds were cut using a vibrating blade microtome (Leica VT1000 S, Leica Microsystèmes SAS, France) in sterile distilled water. Then, sections were quickly put on a glass slide in 100 μl of a Nile Red solution that contained 0.1% Nile Red (w/v) in 50% glycerol. The cell walls were counterstained by adding 100 μl of a Calcofluor solution that contained 1% Fluorescent Brightener 28 (w/v) in a carbonate/bicarbonate buffer pH 9.2. For Nile Red imaging, 488 nm was used for excitation and emission was collected between 593 and 654 nm. For Calcofluor imaging, 405 nm was used for excitation and emission was collected between 412 and 483 nm.

### Metabolome analysis by gas chromatography coupled to mass spectrometry (GC-MS)

For each tissue (i.e., embryo, endosperm), metabolite samples were obtained starting from three replicates of 100 rice seeds manually dissected in embryo and endosperm fractions. 100 embryos and 100 endosperms were grinded with mortar and pestle under liquid nitrogen for the embryos and with a Cyclotec™ 1093 Sample Mill (FOSS, Hillerød, Danemark) for the endosperms. All samples were lyophilized and around 20 mg dry weight (DW) of each sample were placed in 2 ml Safelock Eppendorf tubes (Eppendorf AG, Hamburg, Germany). All analysis steps including extraction, derivatization, analysis, and data processing were adapted from the original protocol described by Fiehn et al. (2008) and following the procedure described by Avila-Ospina et al. (2017). The extraction solvent was prepared by mixing water:acetonitrile:isopropanol at the volume ratio 2:3:3 allowing to extract metabolites with a broad range of polarities. For derivatization step, N-methyl-N-trimethylsilyl-trifluoroacetamide (MSTFA; Sigma-Aldrich) was used in silylation procedure of metabolites. Samples were analyzed on an Agilent 7890A gas chromatograph coupled to an Agilent 5975C mass spectrometer. Raw Agilent datafiles were converted in NetCDF format and analyzed with AMDIS (Automated Mass Deconvolution and Identification System; http://chemdata.nist.gov/mass-spc/amdis/). A home retention indices/mass spectra library built from the NIST, Golm, and Fiehn databases and standard compounds was used for metabolites identification. Peak areas were then determined using the QuanLynx software (Waters, Milford, USA) after conversion of the NetCDF file in MassLynx format.

### RNA isolation and microarray analyses

Total mRNAs were isolated from three replicates of 100 embryos and 50 endosperms and hybridizations on the Affymetrix GeneChip® Rice Genome Array (Affymetrix, Santa Clara, CA, USA) were performed as previously described (Galland et al., 2014a). To obtain presence/absence calls for each probe, we normalized the CEL files by the MAS5 algorithm (Affymetrix). The CEL files were then normalized with the GC-RMA algorithm using the “gcrma” library available from the R Bioconductor suite of open-source softwares (Huber et al., 2015). To determine differentially expressed genes in the embryo and endosperm transcriptomes, we performed a usual two group *t*-test that assumes equal variance between groups. The raw *P*-values were adjusted by the Bonferroni method. We considered a gene as differentially expressed if adjusted-value is < 0.01. To establish the Pearson correlation, we plotted the embryo against the endosperm normalized mean probe intensity. All raw CEL files are available from the Gene Expression Omnibus under the accession GSE43780 (for the embryo: GSM1071216, GSM1071217, GSM1071204; for the endosperm: GSM1071199, GSM1071201, GSM1071210).

### Proteome analysis

#### Protein extraction and in-gel digestion

For embryo protein extraction, three replicates of 50 embryos were ground in liquid nitrogen using mortar and pestle. Then, total soluble proteins were extracted at room temperature in 400 μl thiourea/urea lysis buffer (7 M urea, 2 M thiourea, 6 mM Tris-HCl, 4.2 mM Trizma® base (Sigma-Aldrich, Lyon, France), 4% (w/v) CHAPS) supplemented with 50 μl of the protease inhibitor cocktail Complete Mini (Roche Diagnostics France, Meylan, France). Then, 15 μl of dithiothreitol (DTT, 1 M, Sigma-Aldrich), 2 μl of DNase I (Roche Diagnostics) and 5 μl of RNase A (Sigma-Aldrich) were added to the sample. For endosperm protein extraction, three replicates of 5 endosperms were ground in liquid nitrogen using mortar and pestle. Then, total soluble proteins were extracted at room temperature in 1 ml thiourea/urea lysis buffer (same composition as above) supplemented with 35 μl of DTT, 2 μl DNAseI and 10 μl RNAse A. Finally, protein extracts were let to agitate for 2 h at 4°C. All samples were then centrifuged at 20,000 g at 4°C for 15 min. The resulting supernatant was submitted to a second clarifying centrifugation as above. The final supernatant was kept and protein concentrations in the various extracts were measured according to Bradford (1976) using Bovine Serum Albumin as a standard.

Twenty-five microgram of embryo and endosperm soluble protein extracts (*n* = 3 biological replicates) were subjected to SDS-PAGE analysis with 10% acrylamide (Figure S1). Each lane was systematically cut in 16 bands and submitted to in-gel digestion with the Progest system (Genomic Solution) according to a standard trypsin protocol. Gel pieces were washed twice by successive separate baths of 10% acetic acid, 40% ethanol, and acetonitrile. They were then washed twice with successive baths of 25 mM NH_4_CO_3_ and ACN. Digestion was subsequently performed for 6 h at 37°C with 125 ng of modified trypsin (Promega) dissolved in 20% methanol and 20 mM NH_4_CO_3_. The peptides were extracted successively with 2% trifluoroacetic acid (TFA) and 50% ACN and then with ACN. Peptide extracts were dried in a vacuum centrifuge and suspended in 20 μl of 0.05% TFA, 0.05% HCOOH, and 2% ACN.

#### LC-MS/MS analysis

Peptide separation by NanoLC was performed as described previously (Bonhomme et al., 2012). Eluted peptides were analyzed on-line with a Q-Exactive mass spectrometer (Thermo Electron) using a nano-electrospray interface. Peptide ions were analyzed using Xcalibur 2.1 with the following data-dependent acquisition parameters: a full MS scan covering 300–1,400 range of mass-to-charge ratio (m/z) with a resolution of 70,000 and a MS/MS step (normalized collision energy: 30%; resolution: 17,500). MS/MS Step was reiterated for the 8 major ions detected during full MS scan. Dynamic exclusion was set to 45 s. A database search was performed with X!Tandem (Craig and Beavis, 2004). Enzymatic cleavage was declared as a trypsin with two possible misscleavage. Cys carboxyamidomethylation was set to static modifications. Met oxydation was set as possible modifications. Precursor mass and fragment mass tolerance were 10 ppm and 0.02 Th, respectively. The 7th annotation of the Rice Genome Annotation Project database (Kawahara et al., 2013; 66,338 proteins) and a contaminant database (trypsin, keratins) were used. Only peptides with an *E*-value smaller than 0.1 were reported.

Peptide quantification was performed by extracted ion current (XIC) using MassChroQ software (Valot et al., 2011). A 5 ppm precision windows was set for XIC extraction. We eliminated the peptide ions not specific of a single protein and, since a peptide ion was detected several times in one biological sample, we summed the Total Ionic Current (TIC) area under peak corresponding to the same peptide ion. We also removed peptide ions that were not reliably detectable by keeping only peptide ions detected at least twice out of the three biological replicates. We obtained a final number of 34,179 and 11,824 peptide ions in the embryo and endosperm respectively corresponded to 2,099 and 786 non-redundant proteins. Since several peptide ions corresponded to the same protein, we summed the total peptide ions' TIC area to get the overall protein abundance and we then log_2_-transformed this protein abundance.

### Gene singular enrichment analysis

Gene Singular Enrichment Analysis were performed using the Gene Ontology analysis toolkit provided by the AgriGO web resource (Du et al., 2010) with the Affymetrix (transcriptome) or the corresponding tissue proteome (2099 embryo or 786 endosperm proteins) as backgrounds. The *p*-values generated by a classical hypergeometric overrepresentation test were adjusted by the Yekutieli False Discovery Rate.

### Phylogenetic analysis

The amino acid sequence of the 12 known glutelins and the putative new glutelin, namely Glu-X, were retrieved from the Rice Genome Annotation Project (Ouyang et al., 2007). Protein sequences were aligned with Clustal Omega (Sievers et al., 2011) with allowed gaps and a distance matrix computed (BLOSUM62 matrix). The corresponding phylogenetic tree was built using the Neighbor-joining method.

### Measurement of translational activity

We imbibed three biological replicates of 20 isolated embryos and 5 embryoless endosperms in 4 ml of sterile distilled water with 50 μCi of [^35^S]-Met (PerkinElmer) at 30°C during 24 h in the dark. Samples were placed on filter papers to remove excess water and grinded with mortar and pestle using liquid nitrogen. Proteins were then extracted according to previously published protocols (Rajjou et al., 2006). To avoid measuring the non-specific incorporation of radioisotopes into contaminants, we purified the total soluble proteins. In addition, dead seeds (autoclaved seeds) were used as a negative control in order to measure the background level related to non-specific incorporation of [^35^S]-Met. Finally, 10 μl of protein extracts were added to 5 ml of scintillation liquid cocktail [Ecolite(+), MP Biomedicals, France]. Radioactivity was finally measured (3 biological and 3 technical replicates) using a liquid scintillation analyzer (Tri-Carb 2810TR, PerkinElmer, MA, USA) set between 5 and 100 keV with 10 min integration per sample.

## Results

### Tissue anatomy of the rice mature seed

**Figure 1:**
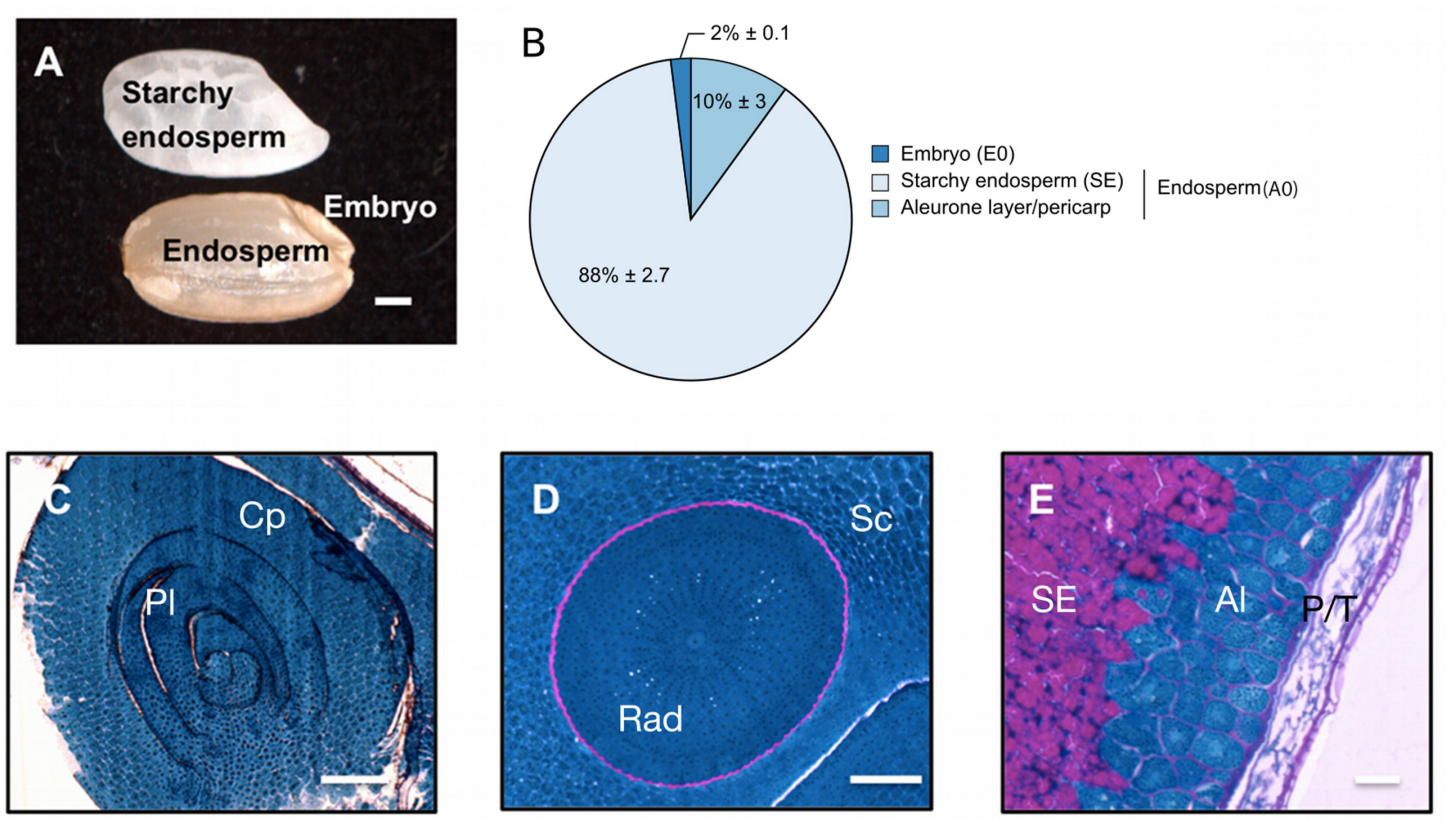
Description of the dry mature rice seed. **(A)** The dry mature rice seed (*Oryza sativa* ssp. *japonica* cv Nipponbare) is composed of several tissues including the embryo, endosperm (pericarp, testa, aleurone layer and starchy endosperm) and inner starchy endosperm. Scale bar, 1 mm. **(B)** Dry weight per seed of the isolated embryo (E0), starchy endosperm and the aleurone layer/pericarp tissue. The average percentage of each seed tissue is indicated (average % per seed) along with its standard-deviation (*n* = 10). The endosperm (A0) is the combination of the starchy endosperm (SE) and of the aleurone layer/pericarp tissue. **(C–E)** Proteins (blue) and complex carbohydrates (including starch, pink) were revealed using a Periodic Acid Schiff—Naphthol Blue Black staining on 5 μm historesin-embedded semi-thick sections. Stained sections of the embryo shoot apical meristem (**C**, longitudinal cut), embryo radicule (**D**, transversal cut) and endosperm dorsal side **(E)** were visualized by optic microscopy. Scale bars represent 100 μm in **(C,D)** and 25 μm in **(E)**. Al, Aleurone layer; Cp, coleoptile; Pl, plumule; P/T, pericarp/testa; Rad, radicle; Sc, scutellum; SE, starchy endosperm.

**Figure 2:**
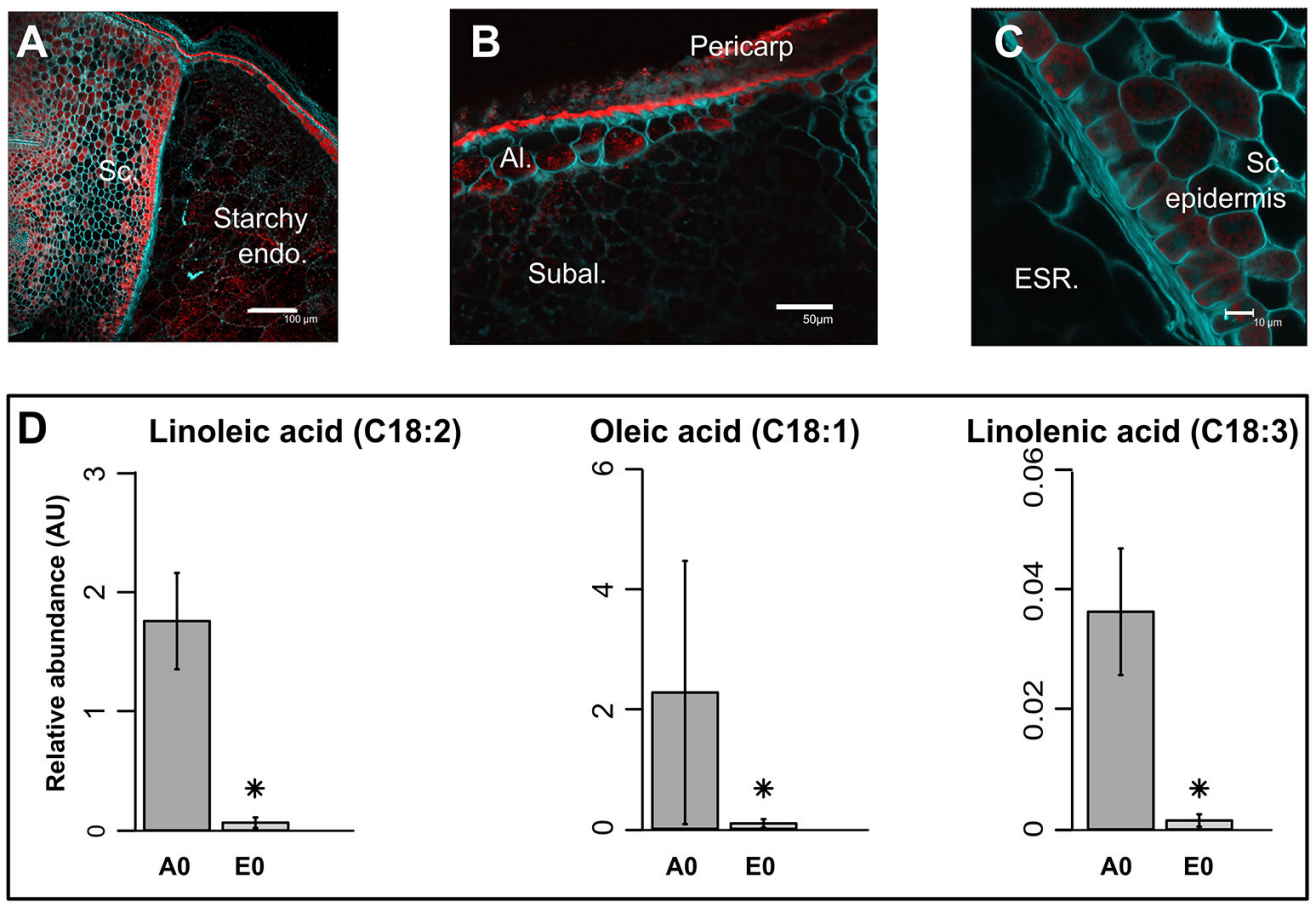
Precise localization of oil bodies and compartmentation of fatty acids between rice seed tissues. **(A–C)** Lipids were visualized by confocal microscopy on fresh 100 μm sections of mature rice seeds. Fresh sections were stained by both Nile Red (red channel) to monitor neutral lipids and Calcofluor (green channel) to reveal cell walls. Scale bars are 100 μm in **(A)**, 50 μm in **(B)**, and 10 μm in **(C)**. Sc, scutellum; Endo, endosperm; Al, aleurone layer; Subal, subaleurone layer; ESR, embryo surrounding region. **(D)** Bar plots represent the mean seed equivalent lipid abundance (*n* = 3). Asterisks indicate significant (*p* < 0.05, False Discovery Rate) different means. A0, endosperm; E0, embryo.

The pericarp, testa and AL were removed using a lab-polishing machine to obtain the inner part of the rice seed i.e., the starchy endosperm (Galland et al., 2014a; Figure 1A). On a dry weight basis, the embryo represents only 2% of the whole dry seed with the endosperm accounting for the remaining 98% (Figure 1B). Within the endosperm, the starchy endosperm represents 87.8% of the endosperm dry weight (Figure 1B). To describe the anatomy and content of the rice seed tissues, 5 μm semi-thin sections of dry seeds were obtained and embedded in resin and stained with specific reagents (Figures 1C–E). The embryo appeared as a very cell-dense tissue rich in proteins with no complex sugars detectable except in cell walls and around the radicle (Figures 1C,D). In contrast, the endosperm appeared as heterogeneous tissue displaying a marked differentiation between the inner starchy endosperm (rich in starch and storage proteins) and the living AL (visible nuclei and numerous protein bodies) (Figure 1E). Lipids were detected by Nile Red tissue staining in both rice endosperm and embryo (Figures 2A–C), with local enrichments in the aleurone/subaleurone layers (Figure 2B) and scutellum epidermis (Figure 2C). These cytological observations show the important degree of compartmentation within the dry mature rice seed.

### Metabolic composition of the rice mature seed compartments

**Figure 3:**
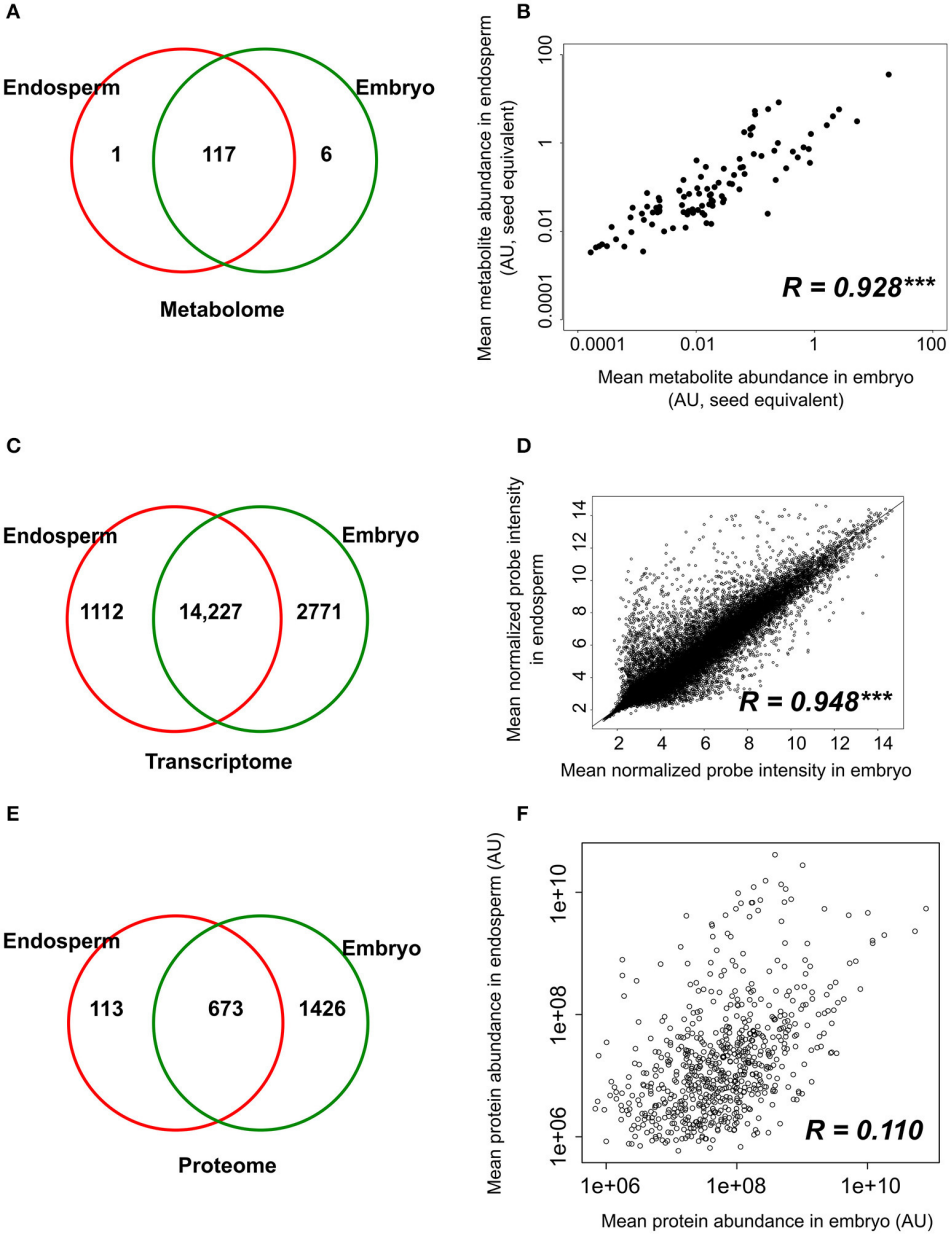
Tissue-specificity and correlations between component tissue molecular apparatus. **(A,C,E)** Venn diagrams of the number of metabolites **(A)**, probe sets **(B)**, and proteins **(C)** detected in the dry embryo and endosperm. **(B,D,F)** Pearson correlations were calculated based on the metabolite level **(B)**, probe signal **(D)** and protein abundance **(F)** in the embryo and endosperm. Protein and metabolites are plotted on a log_2_ and log_10_ scale respectively. Transcript (probe sets) intensities are log_2_ transformed during normalization and are plotted on non-transformed axis. Pearson correlation coefficients are indicated on each graph along with their significance level (^***^p < 0.001).

Metabolomic data were generated from both embryo and endosperm (Table S1). Thus, 124 unique metabolites were identified at least once either in the rice seed embryo or endosperm and most of them (i.e., 117) were detected in all seed compartments (Figure 3A). Indeed, we only identified six embryo-specific metabolites i.e., γ-tocopherol, feruloylquinic acid, maltotriose, adenosine-5-P and two galactinol isomer (m/z equal to 204 and 433, Table 1). In contrast, ascorbate was detected only in the endosperm (Table 1). A quantitative analysis was performed on the 117 common metabolites detected in both seed compartments. The abundances of the metabolites were normalized according to the dry weight of the embryo and endosperm and a differential analysis performed that revealed 72 differentially accumulated metabolites (*p* < 0.05, Table 1). We found a strong correlation between the embryo and endosperm metabolite abundance per seed (Figure 3B). Thus, despite the differential ploïdy and origin of the mature rice seed tissues, the composition in terms of primary metabolites is rather similar on a per seed basis. Nevertheless, most of these metabolites were more abundant in the endosperm such as unsaturated fatty acids: oleic acid (C18:1), linoleic acid (C18:2) and linolenic acid (C18:3) (Figure 2D). Only a few of them (e.g., raffinose, citrate, α-tocopherol, glucaric acid, digalactosylglycerol) were significantly more abundant in the embryo (Table 1).

**Table 1:** Tissue-specific and differentially accumulated metabolites between embryo and endosperm of the dry mature rice seed.

**Compound^a^**	**Class**	**Endosperm/embryo log_2_ ratio^b^**	**FDR^c^**
γ-tocopherol	Apolar	NA	Embryo
Maltotriose	Carbohydrate	NA	Embryo
Ascorbate	Organic acids	NA	Endosperm
Feruoylquinic acid	Organic acids	NA	Embryo
Adenosine-5-P	Purine_Pyrimidine	NA	Embryo
Galactinol_isomer_1	Sugar alcohol	NA	Embryo
Galactinol_isomer_2	Sugar alcohol	NA	Embryo
4-hydroxyproline	Amino acids	1.95	0.05
Alanine	Amino acids	0.88	0.04
beta-Alanine	Amino acids	1.76	0.03
3-cyano-alanine	Amino acids	3.31	0.03
Cystein	Amino acids	1.77	0.05
4-Aminobutyrate (GABA)	Amino acids	2.29	0.01
Glutamine	Amino acids	3.00	0.01
Isoleucine	Amino acids	2.30	0.04
Leucine	Amino acids	2.21	0.03
Lysine	Amino acids	1.32	0.00
Ornithine	Amino acids	0.84	0.03
Phenylalanine	Amino acids	2.57	0.03
Pyroglutamate	Amino acids	0.97	0.03
Serine	Amino acids	2.05	0.03
Threonine	Amino acids	1.59	0.04
Tryptophan	Amino acids	1.20	0.01
Valine	Amino acids	2.39	0.04
α-Tocopherol	Apolar	−2.55	0.03
β-Sitosterol	Apolar	2.57	0.00
Campesterol	Apolar	1.72	0.00
Monopalmitin	Apolar	3.83	0.03
Monostearin	Apolar	4.62	0.02
Stigmasterol	Apolar	3.18	0.00
N-acetylmannosamine	Other	2.49	0.02
Arabinose	Carbohydrate	4.38	0.02
Cellobiose	Carbohydrate	1.95	0.03
Galactose	Carbohydrate	5.64	0.02
Glucopyranose	Carbohydrate	4.30	0.02
Mannose	Carbohydrate	1.54	0.01
Melibiose	Carbohydrate	1.49	0.01
Ribose	Carbohydrate	5.07	0.01
Sedoheptulose	Carbohydrate	3.56	0.02
Trehalose	Carbohydrate	1.53	0.00
Xylose	Carbohydrate	4.42	0.02
Raffinose	Carbohydrate	−0.76	0.02
Sucrose	Carbohydrate	0.98	0.01
Linoleic acid	Fatty acids	4.76	0.01
Linolenic acid	Fatty acids	4.61	0.02
Caffeate	Organic acids	4.73	0.03
Citrate	Organic acids	−1.24	0.01
Erythronate	Organic acids	3.57	0.05
Fumarate	Organic acids	3.20	0.05
4-hydroxybutanoate	Organic acids	5.34	0.01
Glucaric acid	Organic acids	−2.69	0.01
Gluconate	Organic acids	1.96	0.03
Glycerate	Organic acids	3.80	0.03
Glycolate	Organic acids	4.57	0.03
Malate	Organic acids	2.77	0.01
Maleate	Organic acids	3.90	0.01
Malonate	Organic acids	2.35	0.05
Nicotinate	Organic acids	2.11	0.03
Phosphate	Organic acids	1.15	0.01
Pipecolate	Organic acids	0.68	0.01
Salicylate	Organic acids	4.03	0.05
Sinapinate	Organic acids	3.90	0.03
Sinapinate-trans	Organic acids	4.01	0.01
Succinate	Organic acids	3.28	0.03
Threonate	Organic acids	1.50	0.00
Spermidine	Polyamine	2.76	0.01
Guanine	Purine_Pyrimidine	4.31	0.03
Uracil	Purine_Pyrimidine	3.62	0.04
Erythritol	Sugar alcohol	3.59	0.00
Glycerol	Sugar alcohol	4.19	0.03
Glycerol-2-P	Sugar alcohol	1.56	0.01
Glycerol-3-P	Sugar alcohol	0.56	0.01
Inositol_isomer_3	Sugar alcohol	2.15	0.03
Inositol_isomer_4	Sugar alcohol	1.59	0.01
Inositol_isomer_5	Sugar alcohol	2.66	0.03
Inositol-1-P	Sugar alcohol	3.15	0.03
Mannitol	Sugar alcohol	3.98	0.03
Digalactosylglycerol	Sugar alcohol	−0.61	0.00
Galactosylglycerol	Sugar alcohol	4.38	0.03

a See additional file 1 for compound identification details (m/z and retention index)

b *The average seed metabolite abundance in each tissue (endosperm or embryo) was used to calculate the log_2_ ratio. A positive log_2_ ratio indicates an endosperm-favored metabolite accumulation (negative for embryo-favored log_2_ ratio)*.

c *For each metabolite, a Student t-test followed by a False Discovery Rate (FDR) correction was computed. This table contains only metabolites with a p < 0.05. Tissue-specific metabolites are indicated (e.g., “Embryo”)*.

### Analysis of long-lived stored mRNAs in the embryo and endosperm

The transcriptome of both the embryo and endosperm was analyzed using the Affymetrix Rice Genome Array and a dedicated workflow (Table S2, Figure S2A, Jung et al., 2008). Ambiguous and “absent” probe sets were removed which gave a final number of 15,339 and 16,998 detectable probe sets in the endosperm and embryo, respectively (equivalent to 12,964 and 14,150 unique genes representing 33 and 36% of the total genes) (Table S2C). The existence of a large overlap with 14,227 probe sets commonly detected in both endosperm and embryo was highlighted by these data (Figure 3C). Furthermore, a high significant correlation (*R* = 0.948, *p* < 0.001) was found between the normalized probe intensities in the embryo and endosperm transcriptomes (Figure 3D). Yet, some tissue-specific transcripts were detected with 2771 embryo-specific (E0) probe sets (corresponding to 2613 single genes) and 1112 endosperm-specific (A0) probe sets (corresponding to 903 single genes, Figure 3C). Altogether, these results suggest that the endosperm transcriptome is comparable qualitatively and quantitatively to that of the embryo.

The biological roles for the genes that were strongly (superior to median) and differentially regulated (*p* < 0.01) between the endosperm and the embryo were then analyzed, which yielded to 787 and 1,921 probes (corresponding to 728 and 1746 single genes, respectively) that showed a preferential accumulation in the endosperm and embryo, respectively. Among the GO terms enriched in the 787 endosperm-favored probe sets, the “serine-type endopeptidase inhibitor activity” category, which contains known rice allergenic proteins (RAL2-5) associated with α-amylase or trypsin inhibitory functions, was found (AgriGO, Du et al., 2010, *p* < 0.05, Figure S3). Concerning the 1,921 probes with an embryo-favored expression, highly overrepresented GO terms related to “ribosome biogenesis,” “translation,” “rRNA binding,” “ribosomal large and small subunit,” and “structural constituent of ribosome” was detected (Figure S4). It seems therefore that a large proportion of the embryo long-lived mRNAs will serve as the basis for translation, a conserved and essential process for seed germination (Rajjou et al., 2012).

### Proteome analysis of the rice dry mature seed

**Figure 4:**
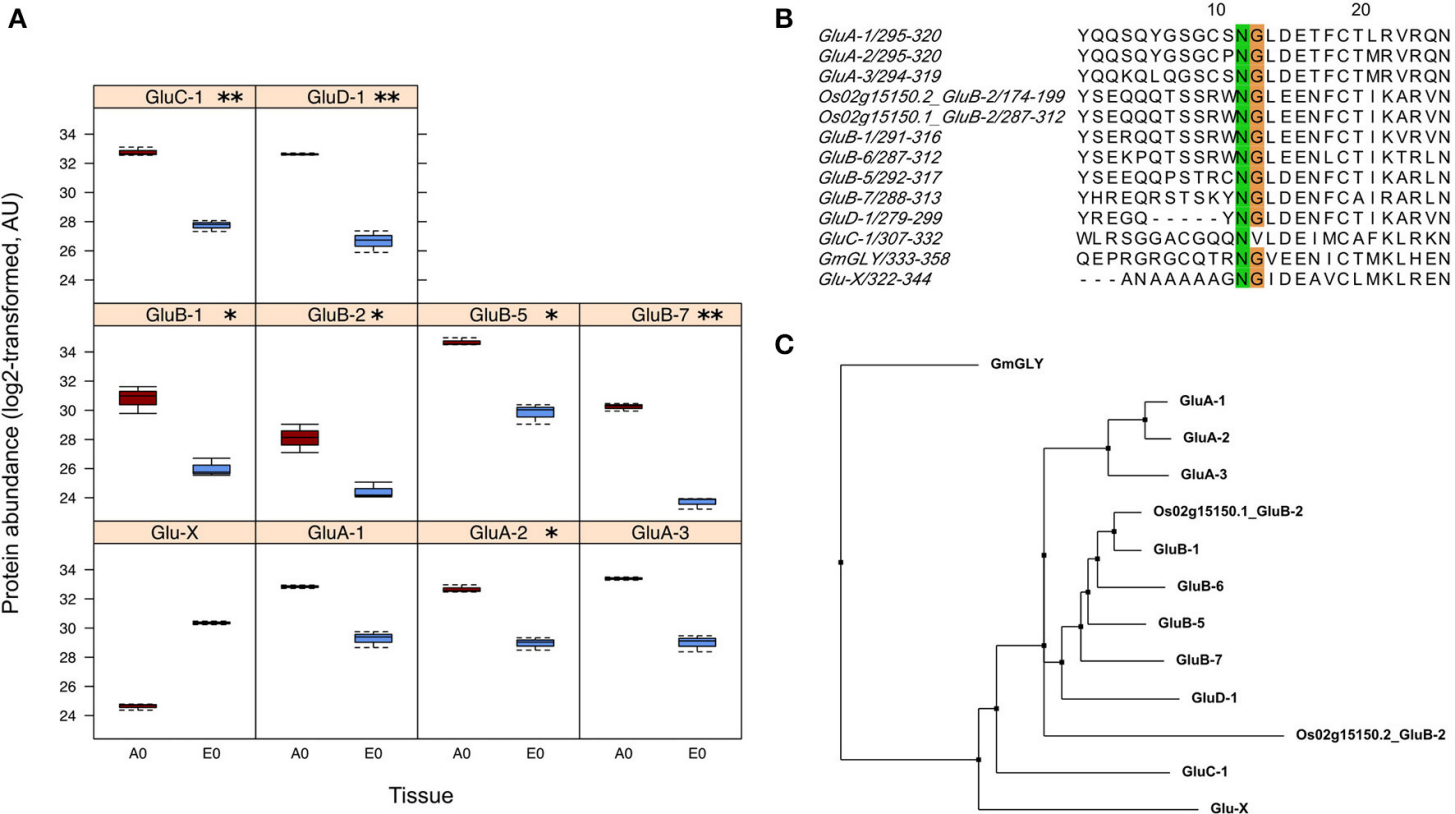
Identification of a putative new glutelin with a preferential embryo accumulation. **(A)** Boxplots of protein abundances (log_2_ transformed) for the 10 glutelins that are commonly detected in the endosperm (A0, dark red) and the embryo (E0, blue). The Glu-X protein corresponds to a newly discovered glutelin which is the only glutelin more abundant in the embryo although not significantly. Significantly different means are indicated according to the z-score analysis with *p*-values inferior to 0.01 (^**^) or 0.05 (^*^). **(B)** Close-up view of the conserved cleavage site of glutelin precursor into acidic and basic subunits. The GluC-1 protein sequence does not exibit the characteristic NG motif. **(C)** Phylogenetic tree built from the 12 glutelin protein sequences together with the newly putative Glutelin, namely Glu-X (neighbor joining, BLOSUM62 matrix)

Regarding seed biology, post-transcriptional and translational regulations add a significant level of complexity as exemplified by studies on developing Arabidopsis and Medicago seeds where numerous examples of delays between mRNA accumulation and protein synthesis have been documented (Gallardo et al., 2007; Hajduch et al., 2010; Verdier et al., 2013). Thus, to complete the description of each rice seed compartment, a proteomic analysis was carried out. The total soluble proteins of embryo and endosperm tissues were extracted and these samples were subjected to a label-free quantitative shotgun proteomic analysis (Figure S1, Figure S2B, S5; Table S3). A total of 2212 single proteins were identified of which only 30.4% (673 proteins) were common to both compartments thereby revealing 1426 embryo-specific and 113 endosperm-specific proteins (Figure 3E). These results showed that the embryo proteome is much more diversified than the endosperm proteome. Furthermore, the abundance of the 673 common proteins is poorly correlated (Figure 3F) contrasting with what was observed at the transcriptome and metabolome levels (Figures 3B,D). For each of the 673 common proteins, the endosperm to embryo protein log_2_ ratio was calculated and we found 76 and 267 proteins with an endosperm-favored or embryo-favored abundance respectively (log_2_ ratios superior to the median i.e., 1.7 and −2.9 for the endosperm and embryo). By combining these proteins showing a tissue-favored profile with the tissue-specific proteins, we obtained a list of 189 endosperm and 1,693 embryo proteins that we subsequently analyzed using the AgriGO tool (Du et al., 2010). In the endosperm, several expected enriched GO terms were retrieved such as “carbohydrate metabolic process” or “plastid” related to starch biosynthesis (Figure S6). In addition, others interesting enriched GO terms such as “response to endogenous stimulus,' “cell wall,” and “vacuole” were highlighted. These functions are probably related to the developed vesicle trafficking occurring during endosperm development and PCD. In the embryo, a strong GO enrichment for biological processes related to “translation,” “embryonic development,” “post-embryonic development” but also “response to stress” was found (Figure S7). At the subcellular level, several key organelles related terms were also overrepresented such as those related to the “mitochondrion,” “plastid,” or “ribosome” (Figure S7). Since the distribution of the log_2_ ratios was similar to a normal distribution (Shapiro-test *p* = 0.95, Figure S5G), a z-score analysis was also performed. This revealed 64 proteins as being differentially accumulated between the embryo and the endosperm (*p* < 0.05; Table S3D). Among these 64 differentially accumulated proteins, 47 are more abundant in the endosperm with several classical proteins classically found enriched in the endosperm such as SSPs (glutelins, prolamins, globulin) and starch biosynthesis enzymes (Table 2). Among the 10 glutelins commonly detected to both seed tissues, all of them were significantly (*p* < 0.05) more abundant in the endosperm (7–13 fold, Figure 4A). An additional protein (Os08g03410, called Glu-X), annotated as a putative glutelin, is preferentially accumulated in the embryo (11 fold, Figure 4A). Glu-X display high sequence homology to other proteins belonging to the glutelin GluA/B/C/D families (Figures 4B,C). Furthermore, we were able to find one eukaryotic translation factor eIF4A-1 or the rice homolog of the MOTHER of FT and TFL1 as two endosperm-favored proteins not directly related to classical endosperm proteins (Table 2).

**Table 2:** Proteins differentially accumulated between endosperm and embryo.

**Protein**	**Description**	**Endosperm to embryo log_2_ ratio^a^**	***p*-value^b^**
Os02g06410.1	Putative SNF-1 related protein kinase	−8.13	0.01
Os12g29400.1	GRAM domain containing protein, expressed	−8.02	0.02
Os05g39250.1	PEBP (phosphatidylethanolamine-binding) family protein	−7.67	0.02
Os05g49440.2	DUF1264 domain containing protein	−7.62	0.02
Os03g15960.1	17.9 kDa heat shock protein	−7.49	0.02
Os03g14180.1	26.7 kDa heat shock protein	−7.47	0.03
Os02g32860.1	Poly [ADP-ribose] polymerase 3	−7.46	0.03
Os03g19290.1	Chloroplastic outer enveloppe pore protein	−7.42	0.03
Os03g04410.1	Aconitase	−7.41	0.03
Os05g37330.1	60S acidic ribosomal protein	−7.38	0.03
Os02g51750.1	Annexin	−7.32	0.03
Os10g30150.1	Universal stress protein	−7.22	0.03
Os03g06360.1	Late embryogenesis abundant protein D-34	−7.18	0.03
Os01g50910.1	Late embryogenesis abundant protein LEA_4 domain	−6.87	0.04
Os05g44340.1	Chaperone protein ClpB1, heat shock protein 101	−6.86	0.04
Os08g41390.1	Putative 70 kDa peptidylprolyl isomerase	−6.74	0.04
Os10g17280.1	ATP synthase gamma chain	−6.71	0.04
Os07g42490.1	Sucrose synthase 3	8.80	0.00
Os07g11630.1	LTPL163 LTP family protein	7.94	0.00
Os12g14070.1	70 kDa heat shock-related protein	7.93	0.00
Os05g26350.1	13 kDa prolamin PROLM4	6.93	0.00
Os05g41970.1	19 kDa globulin	6.74	0.00
Os11g47520.1	Xylanase inhibitor protein 2	6.74	0.00
Os06g09450.1	Sucrose synthase 4	6.62	0.00
Os02g14600.1	Glutelin GluB-7	6.53	0.00
Os02g32660.1	Starch branching enzyme	6.53	0.00
Os07g11410.1	Seed allergenic protein RAG2	6.19	0.00
Os01g44220.1	ADP-glucose pyrophosphorylase	6.08	0.00
Os06g51084.1	Starch branching enzyme 1	6.07	0.00
Os07g10570.1	13 kDa prolamin PROLM25	5.98	0.00
Os02g15090.1	Glutelin GluD-1	5.84	0.00
Os06g04200.1	Granule-bound starch synthase 1 (Waxy)	5.81	0.00
Os05g33570.1	PPDK1 Pyruvate phosphate dikinase 1	5.79	0.00
Os07g11650.1	LTPL164 LTP family protein	5.46	0.01
Os07g11380.1	Seed allergenic protein RAG2	5.33	0.01
Os07g10580.1	13 kDa prolamin PROLM26	5.29	0.01
Os07g11360.1	Seed allergenic protein	5.27	0.01
Os06g22060.1	6-phosphofructokinase pyrophosphate dependent	5.11	0.01
Os02g25640.1	Glutelin GluC-1	5.03	0.01
Os02g15169.1	Glutelin GluB-1b	4.88	0.01
Os07g11510.1	Seed allergenic protein	4.82	0.01
Os07g34520.1	Isocitrate lyase	4.79	0.01
Os02g16820.1	Glutelin GluB-5	4.77	0.01
Os07g11330.1	Seed allergenic protein	4.68	0.01
Os04g40660.1	MA3 domain containing protein	4.59	0.01
Os06g31070.1	13 kDa prolamin PROLM24	4.57	0.01
Os03g55090.1	Starch phosphorylase	4.54	0.01
Os03g16440.1	Translocon at outer-enveloppe membrane of chloroplast	4.51	0.01
Os03g31360.1	Glutelin GluA-3	4.34	0.02
Os04g33150.1	desiccation-related protein	4.14	0.02
Os10g35010.1	Translocon at inner-enveloppe membrane of chloroplast	4.00	0.02
Os02g15150.1	Glutelin GluB-2	3.79	0.03
Os10g26060.1	Glutelin GluA-2	3.69	0.03
Os06g48750.1	eIF4A-1 Eukaryotic translation initiation factor 4A-1	3.56	0.03
Os02g50350.1	Dihydropyrimidine dehydrogenase	3.55	0.03
Os01g55690.1	Glutelin GluA-1	3.51	0.03
Os11g40530.1	LTPL162 LTP family protein	3.44	0.03
Os07g11900.1	13 kDa prolamin PROLM19	3.30	0.04
Os01g47410.1	Aspartic proteinase oryzasin-1	3.24	0.04
Os06g46284.1	Probable alpha-glucosidase	3.15	0.04
Os06g30370.1	OsMFT1 homologous to Mother of FT and TFL1	3.08	0.05
Os07g11310.1	LTPL166 LTP family protein	3.06	0.05
Os05g02060.1	Chloroplastic outer envelope pore protein	3.03	0.05
Os02g32030.1	Elongation factor	3.00	0.05

a *The average seed protein abundance in each tissue (endosperm or embryo) was used to calculate the log_2_ ratio. A positive log_2_ ratio indicates an endosperm-favored metabolite accumulation (negative for embryo-favored log_2_ ratio)*.

b *P-value obtained from z-score analysis of endosperm to embryo log_2_ ratios*.

### Identification of post-transcriptional and translational regulations in the mature seed

**Figure 5:**
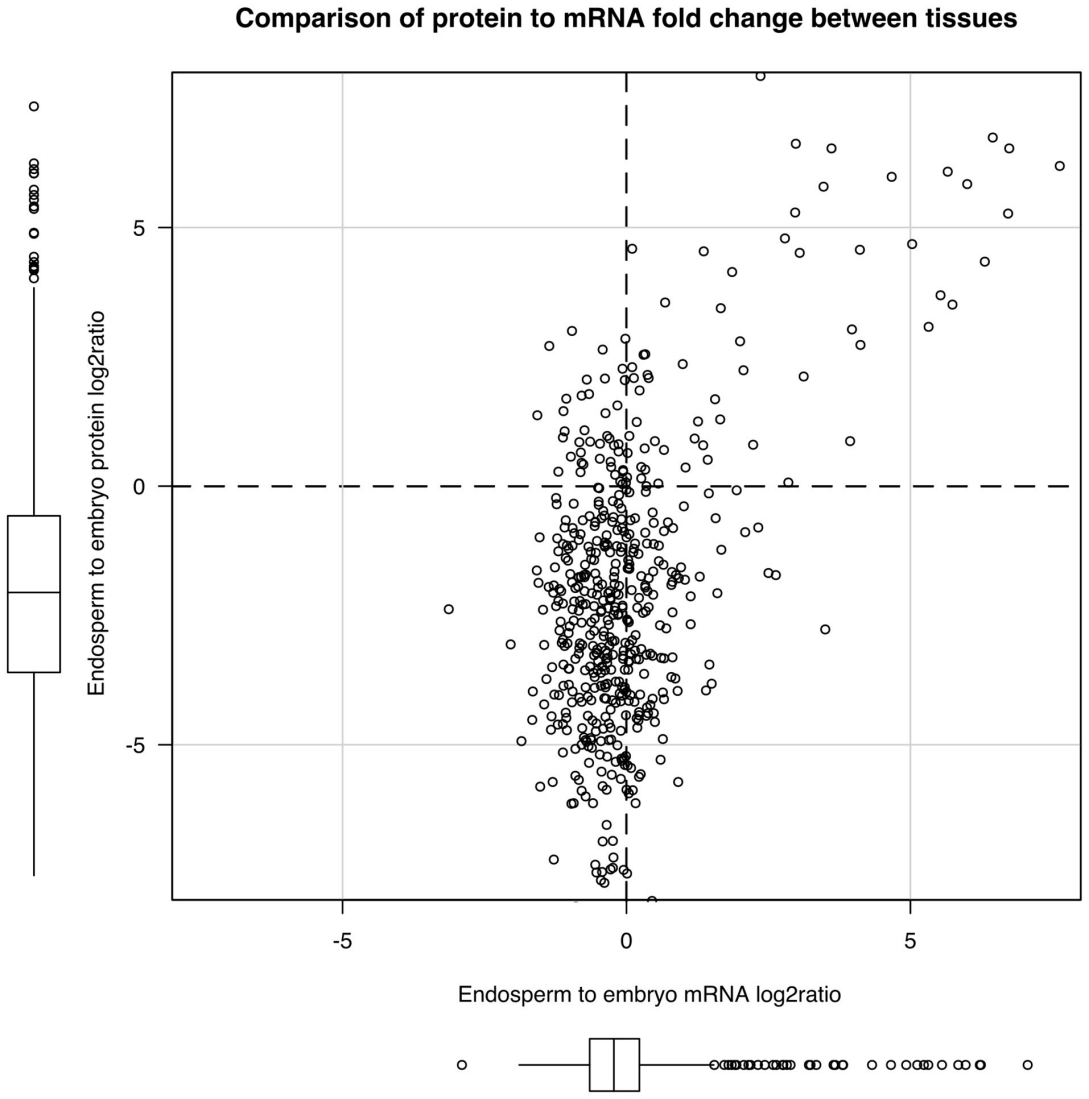
Post-transcriptional regulations in the dry rice mature seed. By keeping unique mRNA-protein pairs among the 673 proteins common to both endosperm and embryo, we obtained 504 pairs. We plotted the mRNA to protein endosperm to embryo log_2_ ratio to display post-transcriptional regulations.

The strong seed tissue differentiation observed at the proteome level could be well connected to the delay between transcript and protein accumulation observed at the end of seed development (Gallardo et al., 2007; Hajduch et al., 2010; Arc et al., 2011; Verdier et al., 2013). We quantitatively compared the 673 proteins fold-change between seed tissues with that of their corresponding mRNA level. These 673 proteins matched with 672 non-redundant genes that were used to retrieve their corresponding probe sets among the 14,227 common probe sets (Figure 3C). The probe sets corresponding to the same gene were removed leading to 504 unique probe set/protein pairs (Table S4). Then, the endosperm to embryo log_2_ ratios of these 504 probe set/protein pairs were compared (Figure 5). From this analysis, it was obvious that the majority of the 504 protein log_2_ ratios were poorly correlated to their corresponding mRNA log_2_ ratio. On the one hand, proteins with an endosperm-favored log_2_ ratio (>0) also have similar endosperm-favored mRNA log_2_ ratios. On the other hand, the proteins favorably accumulated in the embryo (log_2_ ratio < 0) show poorly concordant mRNA profiles. These results once again highlight the seed as a tissue with major strong post-transcriptional and translational regulations probably related to the presence of long-lived mRNAs and to the seed metabolism being brought to a halt (Galland and Rajjou, 2015).

### Measurement of translational activities from isolated embryos and embryoless endosperms

**Figure 6:**
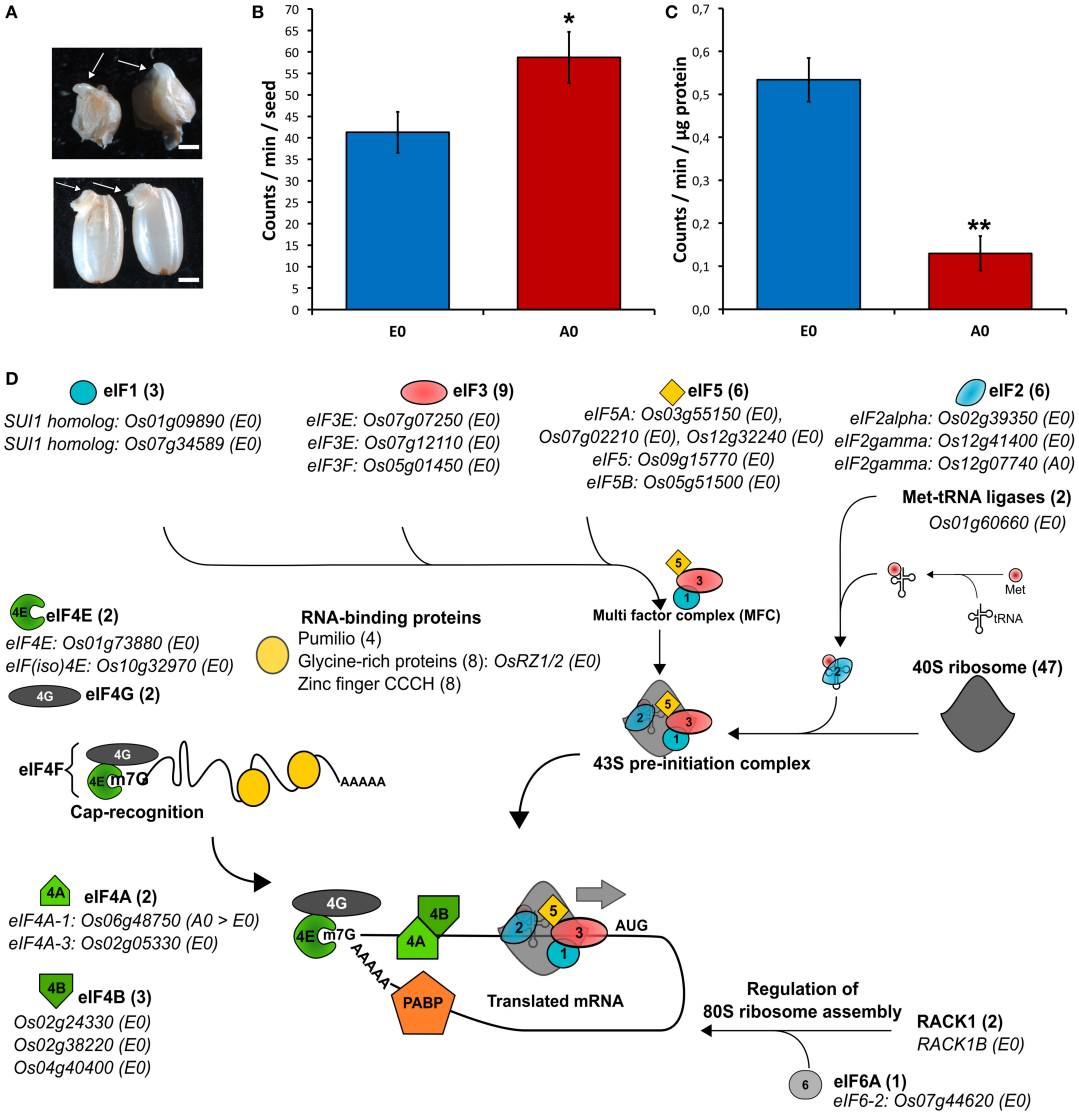
Translational activity and machinery in rice seed tissues. **(A–C)** Translational activities in isolated embryos and embryoless endosperms during germination *sensu stricto*. **(A)** Germination of isolated rice embryos (top panel) or complete seeds (lower panel) as evidenced by coleoptile emergence (arrows). Scale bars represent 2 mm. **(B,C)** Radiolabeled [^35^S]-Methionine incorporation during 24 h of imbibition in 20 isolated embryos (E0) or five embryoless endosperm (A0). **(B)** [^35^S]-Methionine incorporation per seed in isolated embryo (E0) and embryoless endosperm (A0). **(C)** [^35^S]-Methionine incorporation per microgram of total protein in E0 and A0. Results are the mean (± SD) of three biological replicates and are expressed on a seed equivalent basis for comparison. Signal integration was performed during 10 min. Student's *t*-tests were applied to identify statistically significant differences (^*^ means statistically significant as *P* < 0.05 and ^**^ means statistically highly significant as *P* < 0.01). **(D)** Embryo and endosperm proteins involved in translation initiation identified in the dry mature rice seed. For each protein family, the number of proteins found in the rice seed proteome is indicated. Embryo-specific proteins (E0) are indicated along with their locus number. eIF, eukaryotic translation initiation factor; Met, methionine; PABP, polyadenylate-binding protein.

During cereal seed germination, both embryo and endosperm tissues actively transcribe RNA and translate both stored mRNAs and *de novo* synthetized mRNAs (Fincher, 1989). The presently used rice seeds exhibited high vigor (T_50_ = 16 HAI; G_max_ = 24 HAI). The isolated rice embryos were also capable of germination as evidenced by coleoptile emergence upon 24 h of imbibition (Figure 6A). Thus, the translational activities from isolated embryos and embryoless endosperms were assessed. As known for decades, the cereal embryo is an efficient system for *in vitro* translation of mRNAs (Takai and Endo, 2010) while the aleurone layer is actively synthetizing starch-degrading enzymes during germination (Fincher, 1989). Accordingly, considering a single seed, the endosperm had a slightly higher significant translational activity by comparison to the embryo (Figure 6B). By contrast, relatively to total protein content, translational activity is significantly higher in the embryo than in the endosperm (Figure 6C). This result indicates that protein synthesis is more active in the embryo. In support of this statement, the proteomic data revealed that the embryo is better equipped with proteins involved in the translation initiation machinery compared with the endosperm (Figure 6D).

## Discussion

### Origin and roles of the endosperm, a key tissue in seed biology

The supernumerary embryo origin of the endosperm is backed by several results in our study and that of others. As previously observed in the case of developing (Belmonte et al., 2013) or germinating Arabidopsis seeds (Penfield et al., 2006), the transcriptomes of the rice embryo and endosperm are highly similar both quantitatively and quantitatively (Figures 3C,D). In developing maize kernels (8 days after pollination), tissue-specific transcriptomics showed that the embryo transcriptome resembled that of aleurone cell layers but also that of other endosperm regions as Spearman correlations ranged from 0.73 (central starchy endosperm) to 0.80 (aleurone layer) (Zhan et al., 2015). In this latter study, the observed total number of maize endosperm-specific expressed genes (3140) is in the same order of magnitude as ours (1112; Figure 3C). Similar conclusions can be drawn in for the embryo (2235 vs. 2771). Embryo and endosperm transcriptomes in developing and mature cereal caryopses could therefore be quite comparable to each other.

### Stored reserves, building blocks, and energy source for germination

#### Starch

In rice, starch can account for nearly 70–85% of the total seed weight and is made in different proportions of linear amylose and ramified amylopectin (Bao et al., 2008). Historically, the degradation of starch during germination has been considered as a key mechanism that produced the oligosaccharides required for energy production during germination (Fincher, 1989). Here, the AMY3E α-amylase protein (Os08g36900) was among the most abundant embryo-specific proteins (Table S3C) while a probe signal for the *AMY3E* mRNA was detectable in both embryo and endosperm (Table S2B). It is worth noting that no amylase activity could be measured in dry rice seeds (Guglielminetti et al., 1995) suggesting that the preformed embryo AMY3E enzyme is not functional at this stage. In the endosperm, only one α-amylase protein was detected (Os01g51754) mildly ortholog of the *Vigna mungo* AMY1.1 enzyme (Table S3C). Finally, as reported before, no β-amylase protein was detected in rice seed compartments. Still, many β-amylase transcripts were found also suggesting that post-transcriptional and/or translational regulation is likely to occur for these enzymes.

**Figure 7:**
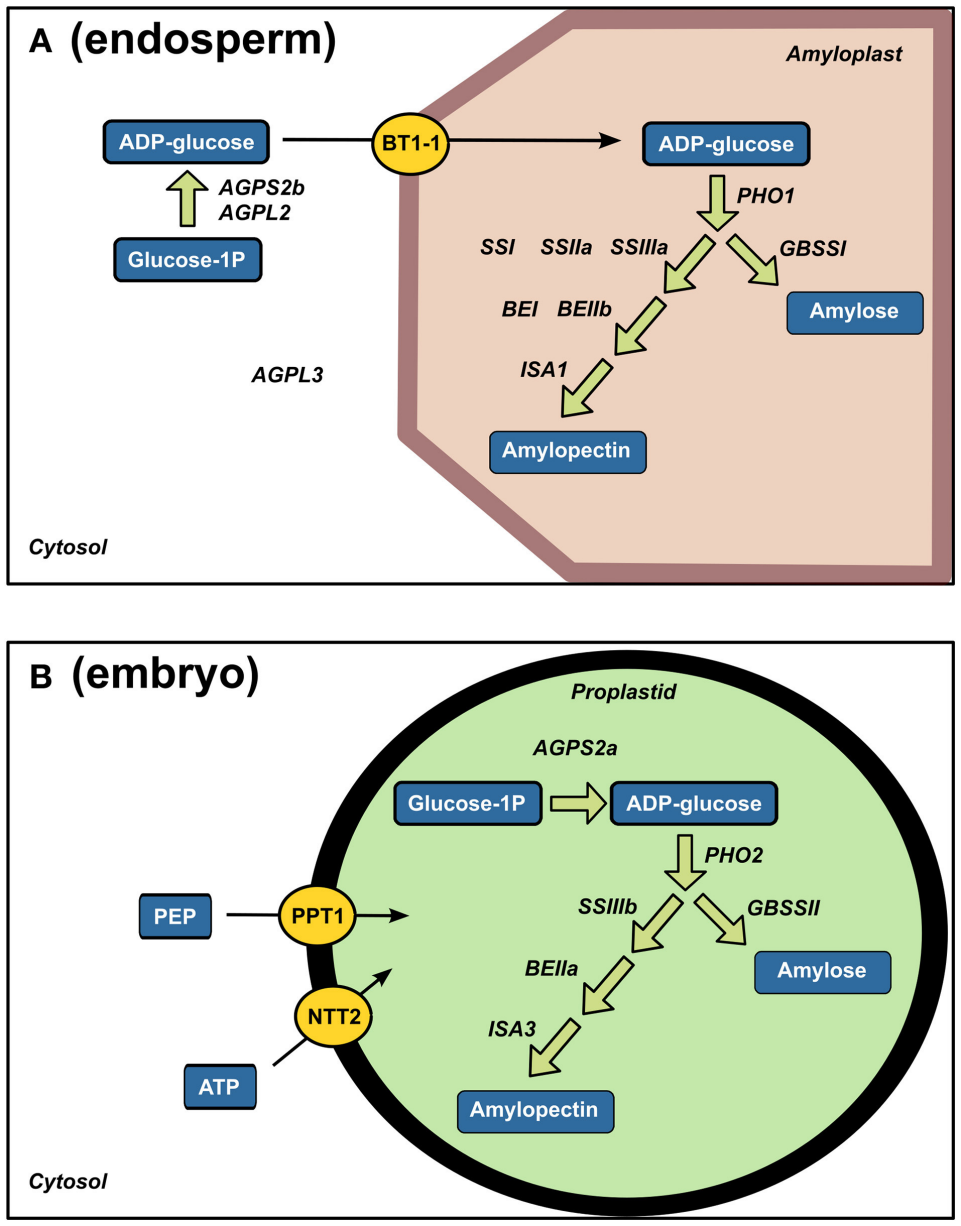
Strong tissue-specificity of starch biosynthesis enzymes between endosperm and embryo. Specific endosperm **(A)** and embryo **(B)** proteins related to starch biosynthesis are displayed on their corresponding enzymatic reactions. AGPL, glucose-1-phosphate adenyltransferase large subunit; AGPS, glucose-1-phosphate adenyltransferase small subunit; BT1-1, brittle 1; PHO, Starch phosphorylase; SS, soluble starch synthase; BE, branching enzyme; ISA, isoamylase; GBSS, granule-bound starch synthase; PEP, phosphoenolpyruvate, PPT, phosphoenolpyruvate/phosphate translocator; NTT, ATP carrier protein.

Along with the presence of few starch-degrading enzymes, a number of starch biosynthesis enzymes were identified (Figure 7A). Starch biosynthesis originates from glucose-1-phosphate that is converted to ADP-glucose by ADP-glucose pyrophosphorylases (AGPases). ADP-glucose serves for α-glucan chain elongation by starch synthases with participation of starch branching enzymes and starch debranching enzymes (Jeon et al., 2010). Most of the studies on rice starch biosynthesis have focused on the endosperm. Accordingly, a complete enzymatic machinery for starch biosynthesis and ADP-glucose transport (OsBT1-1) was detected in the endosperm (Figure 7A). While the presence of these enzymes is not surprising, the present work highlighted a complete starch synthesis enzymatic set already present in the quiescent embryo i.e., AGPS2a, SSIIIb, GBSSII and Pho2 indicating that plastid is the main site of embryonic ADP-glucose synthesis (Figure 7B). In contrast, as a characteristic feature of graminaceous, ADP-glucose synthesis can occurs in the endosperm thanks to a cytosolic ADP-glucose pyrophosphorylase (Beckles et al., 2001). Our results reveal a molecular specificity of each rice seed compartment for starch biosynthesis possibly related to starch synthesis in the embryo during germination (Han et al., 2014).

#### Seed storage proteins (SSPs)

Glutelins are the major SSPs accumulated in rice corresponding to approximately 60–80% of the total proteins in the endosperm. During germination, these proteins are mobilized by proteases hereby releasing free amino acids that can readily be incorporated into new proteins requested for germination. Based on amino acid sequence similarity, previous studies established that 12 genes classified into GluA, GluB, GluC, and GluD families encode for rice glutelins (Kawakatsu et al., 2008). The present proteomic data evidenced 11 glutelin forms belonging to the GluA/B/C/D classification (Table S3C). Since the endosperm was separated from the embryo, it is remarkable to note that only two glutelin isoforms belonging to GluB class (i.e., Os02g15150.2 and Os02g15070.1) were specific of this storage tissue (Table S3C). In agreement with a storage function of the endosperm, among the 10 glutelins common to both seed tissues, all glutelins were significantly (*p* < 0.05) more abundant in the endosperm (7–13 fold, Figure 4A). Unexpectedly, one protein (Os08g03410), annotated as a putative glutelin, is strongly accumulated in the embryo (11 fold, Figure 4A). This protein, hereby named Glu-X, is not classified in the glutelin GluA/B/C/D families but nevertheless presents the characteristic asparagine/glycine (NG) cleavage site specifically recognized by the vacuole-processing enzyme OsVPE (Wang et al., 2009) to process glutelin precursors into the corresponding acidic and basic subunits (Figure 4B; Kumamaru et al., 2010). This Glu-X protein is not closely related to the other glutelin families and could have a distinctive function in the embryo (Figure 4C). For instance, in Arabidopsis, cruciferins seed storage proteins protect the embryo from oxidative stress during seed aging (Rajjou et al., 2008; Nguyen et al., 2015).

#### Lipids

In most cereal seeds, lipids generally account for only 2–3% of the dry weight (Barthole et al., 2012). In seeds, lipids are present in the form of TAGs that are stored in oil bodies corresponding to small vesicles composed of an inner TAG core surrounded by lipid monolayer containing dedicated proteins such as oleosins, caleosins and steroleosins (Murphy, 1993; Murphy et al., 2001; Jolivet et al., 2009). Lipids were detected in both rice endosperm and embryo with local enrichments in the aleurone/subaleurone layers (Figure 2B) and scutellum epidermis (Figure 2C). This precise localization is very similar to that of barley (Neuberger et al., 2008). Yet, the composition of unsaturated fatty acids such as oleic acid (C18:1), linoleic acid (C18:2) and linolenic acid (C18:3) were more abundant in the endosperm (Figure 2D).

**Figure 8:**
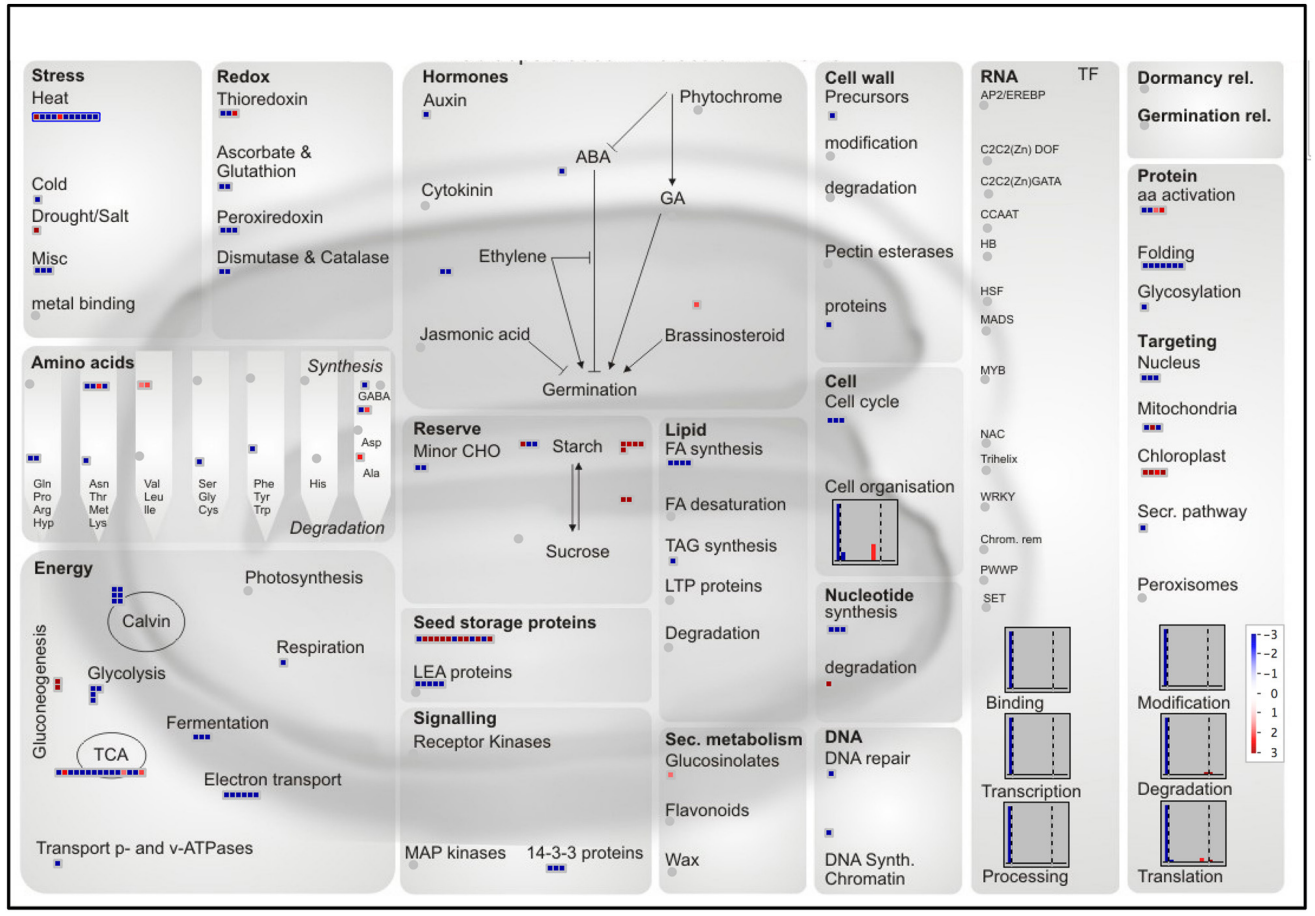
MapMan overview of differentially accumulated proteins with a preferential abundance in the endosperm or embryo. The log_2_ ratios of the 335 proteins with a log_2_ ratio superior to the median were mapped to a seed specific visualization (Joosen et al., 2011). A total of 241 proteins are visible. Red and blue colors represent endosperm and embryo-favored protein abundance respectively.

The nutritional and organoleptic quality of the rice seed is highly dependent on polyunsaturated fatty acid peroxidation caused by lipoxygenases (LOXs). The Aldo-Keto Reductases (AKRs) protein family detoxifies a wide variety of lipid peroxidation compounds. Correspondingly, overexpression of Aldo-ketoreductase-1 from *Pseudomonas* strain (PsAKR1) in rice improved seed viability and germination vigor (Narayana et al., 2017). Two AKRs were specifically present in the embryo (Os04g26910 and Os05g38230) while one AKR was common to both embryo and endosperm (Os01g43090). Plant AKRs were recently proposed as potential breeding targets for developing stress tolerant varieties (Sengupta et al., 2015). In our proteomic data, we detected two rice lipoxygenases (OsLOX2, Os03g52860; OsLOX3, Os03g49350) specifically in the dry embryo in accordance with ancient biochemical results (Table S3C; Ida et al., 1983). Functional analysis showed that OsLOX2 and OsLOX3 negatively affect the germination performance of seeds submitted to artificial or natural aging (Huang et al., 2014; Xu et al., 2015). The higher occurrence of lipid hydroperoxides in aged seeds has been linked with a decrease in seed longevity (Sattler et al., 2004). In addition, the overexpression or silencing of *OsLOX2* accelerates or slows germination *sensu stricto* (Huang et al., 2014). Rice *LOX2* gene expression is induced upon germination presumably to degrade TAGs present in oil bodies and fuel seedling establishment. Therefore, TAGs that are stored in the AL oil bodies would release free fatty acids, fuel carbohydrate synthesis and energy metabolism in the AL and ETC cells during rice seed germination. It has been shown that TAG degradation occurs very early in the embryo and AL cells during the germination process (Clarke et al., 1983; Leonova et al., 2010). Following that hypothesis, the present work reveals in an unexpected way that several mRNAs encoding for glyoxylate cycle enzymes i.e., glyoxysomal malate dehydrogenase (MDH, Os12g43630 and Os05g50940), malate synthase (MLS, Os04g40990) and isocitrate lyase (ICL, Os07g34520) were more abundant in the rice endosperm (Table S2). Notably, ICL was also found as endosperm-favored at the protein level (Table 2). These glyoxylate cycle enzymes preferentially found in the rice endosperm might be associated with anoxia and stressful conditions (Lu et al., 2005). This would also explain the endosperm-favored accumulated of the pyruvate phosphate dikinase 1 (PPDK1, Table 2). In contrast, at the protein level, the rice embryo appears favorably equipped with enzymes involved in glycolysis, tricarboxylic acid cycle and ATP synthesis (Figure 8). The degradation of membrane phospholipids during seed storage is also detrimental to seed quality (Devaiah et al., 2007). In particular, phospholipase D (PLD) enzymes that cleave membrane phospholipids to phosphatidic acid (PA) are proposed to be one the earliest event of deterioration. In Arabidopsis, silencing of the most abundant PLD enzyme, phospholipase D alpha 1 (AtPLDα1), improves seed longevity (Devaiah et al., 2007). Among the 17 *phospholipase D* (*PLD*) rice genes (Li et al., 2007), the *PLD*α*1* (*OsPLD*α*1*, Os01g07760) is the only one expressed and the corresponding enzyme is among the most abundant embryo-specific proteins (Table S3C). Among all rice PLD proteins, OsPLDα1 is closest relative of AtPLDα1 suggesting that their negative roles on seed longevity are probably conserved. In conclusion, the genetic manipulation of TAGs and phospholipid-related enzymes, in particular OsLOX2 and OsPLDα1, present in the rice embryo, have the potential to improve rice seed storability and organoleptic value.

### A tissue-specific equipment possibly involved in desiccation tolerance and seed storability

#### Glassy state establishment

Historically, rice seed longevity has been strongly linked to desiccation tolerance (Ellis and Hong, 1994). Among the mechanisms involved, the accumulation of non-reducing sugars (sucrose, trehalose) and raffinose family oligosaccharides (RFO) at the end of seed development converts the cellular cytoplasm into a “glassy state” that restricts molecule mobility and halt enzymatic reactions (Buitink and Leprince, 2004; Rajjou and Debeaujon, 2008; Farrant and Moore, 2011; Hand et al., 2011). RFO are galactosyl-sucrose carbohydrates that are formed by the sequential addition of galactose moieties by galactinol synthase, raffinose synthase, and stachyose synthase. In the present metabolomic data, sucrose, fructose, glucose, and raffinose were the major simple carbohydrates detected in the dry mature rice seed (Table 1 and Table S1). Furthermore, raffinose is preferentially accumulated in the embryo while non-RFO carbohydrates such as sucrose and trehalose are more highly accumulated in the endosperm (Table 1). Interestingly, a colocalization between QTL of longevity and QTL controlling oligosaccharide contents (sucrose, raffinose, and stachyose) has been pointed out in Arabidopsis and rice (Bentsink et al., 2000; Zhu et al., 2007). Together with raffinose, trehalose can also protect proteins and membranes damages induced by desiccation (Fernandez et al., 2010). It was recently found that the trehalose-6-phosphate phosphatase 7 (Os09g20390, *OsTPP7*) allele from an Indica cultivar was likely to be the underlying QTL for enhanced seed longevity in two Nipponbare near-isogenic lines (Sasaki et al., 2015). The rice genome harbors at least nine trehalose-6-phosphate synthase (*OsTPS*) and nine *OsTPP* genes (Fernandez et al., 2010). One natural candidate for favored trehalose synthesis in the endosperm aleurone layer could be OsTPP10 (Os07g30160) whose transcript is only reliably detected in the endosperm (Table S2E) whereas OsTPP8 protein (Os05g50940) was only detected in the embryo (Table S3C). Thus, the present work provides novel knowledge on the spatial regulation of genes involved in trehalose accumulation in rice seed and possibly related to desiccation tolerance and seed longevity. Thus, in the rice endosperm and aleurone layer in particular, the glassy state seems to be dependent on trehalose, sucrose and raffinose while in the embryo, it depends mostly on raffinose (Table 1).

#### Protein folding protection by molecular chaperones

From the mapping of preferentially accumulated proteins, seed categories related to heat stress, protein folding and LEA proteins were quite noticeable (Figure 8). These categories contain protein chaperone roles such as the LEA proteins, (Wang et al., 2007; Hincha and Thalhammer, 2012), the small HSP (Sarkar et al., 2009; Waters, 2013), annexins (Clark et al., 2012), lipocalins (Grzyb et al., 2006), and ClpB chaperones. Most of these proteins were described to be involved in the maintenance of protein folding, prevent membrane aggregation and can also have a synergic effect with non-reducing sugars and RFO to promote glassy state establishment (Boudet et al., 2006; Rajjou and Debeaujon, 2008; Hand et al., 2011). Commonly related with desiccation and abiotic stress tolerance, LEA proteins are members of intrinsically disordered proteins in aqueous solution. They undergo desiccation-induced folding during cell drying suggesting that these proteins could carry out distinct functions under different water states. Rice comprises 34 LEA proteins encoding genes (Wang et al., 2007). Our proteomic analysis identified 12 LEA proteins detected in both embryo and endosperm and 9 only detected in the embryo (Table 3). LEA proteins were previously associated to seed longevity (Chatelain et al., 2012). It was remarkable to note that several LEA proteins, including the dehydrin family were detected exclusively in the embryo, and could be involved in dry storage survival (Table 3). Indeed, a previous study showed that dehydrin RAB18 was very abundant in Arabidopsis dry mature seeds. The abundance of this protein progressively disappeared in aged seeds (Rajjou et al., 2008). Furthermore, it has been demonstrated that down-regulation of seed-specific dehydrins reduced Arabidopsis seed survival in the dry state (Hundertmark et al., 2011). Out of the 23 predicted sHSP proteins (Sarkar et al., 2009), the HSP17.4 was exclusively found in the endosperm (Table 3). This contrasts with the eight sHSP proteins exclusively found in the embryo of which the OsHSP18.2 (Os01g08860) is capable of protecting the Arabidopsis embryo during artificial aging (Kaur et al., 2015). Furthermore, all three common sHSP proteins (HSP16.9, HSP17.9 and HSP26.7) are preferentially more accumulated in the embryo (Table 3). Remarkably, the HSP16.9 protein was shown to stabilize rice soluble proteins from heat denaturation under *in vitro* conditions (Yeh et al., 1995). Altogether, these results support the finding that such proteins would primarily serve to protect the embryo against desiccation injuries during late maturation program. The present proteomic analysis also revealed several other categories of chaperone proteins such as annexins, lipocalins, and Clp (caseinolytic protease) chaperones which have never been characterized in cereal dry seeds. First, annexins are probably essential for seed longevity since the overexpression of a sacred lotus (*Nelumbo nucifera*) isofom in Arabidopsis proved to enhance seed viability under heat stress (Chu et al., 2012). In the present proteomic data, three annexins with one exclusively present in the embryo (Os09g23160) and one significantly more accumulated in the embryo (Os02g51750, *p* < 0.05) were detected in the present study (Table 3). The last annexin (Os06g11800) was reported to be up-accumulated during germination suggesting a possible role on the embryo membrane dynamics (Yang et al., 2007). Secondly, lipocalins, a family of proteins that transport small hydrophobic molecules such as steroids, bilins, retinoids, and lipids, are classified in plants as temperature-induced lipocalins (TILs) and chloroplastic lipocalins (CHLs) (Charron et al., 2005). It has been demonstrated that both TILs and CHLs are involved in lipid protection, which is critical for stress adaptation. Two TIL proteins are predicted from the rice genome sequence (Charron et al., 2005) and they were detected in the mature rice embryo while the plastidial form OsCHL was undetectable in this tissue (Table 3). These results on the relative abundance of the OsTILs and OsCHL are consistent with those showed in Arabidopsis since the accumulation of AtCHL protein in the AtTIL KO mutant and *vice versa* suggests a functional overlap between these two lipocalin types (Boca et al., 2013). Interestingly, seed longevity is correlated with the accumulation of these proteins in Arabidopsis (Boca et al., 2013).

**Table 3:** Proteins involved in folding and chaperone functions found in the embryo and endosperm.

**Protein**	**Family**	**Description**	**Specificity**	**Rank^a^**	**Endosperm to embryo log_2_ ratio^b^**
Os08g36150.1	ASA1	Heat shock protein 90 co-chaperone	Common	-	−3.9
Os05g44340.1	clpA/clpB	Chaperone protein ClpB1	Common	-	−6.9
Os10g42439.1	DnaJ	DnaJ homolog	Embryo	1,312	-
Os01g04370.1	HSP20	16.9 kDa class I heat shock protein 1	Common	-	−5.4
Os01g08860.1	HSP20	18.0 kDa class II heat shock protein	Embryo	74	-
Os02g52150.1	HSP20	24.1 kDa heat shock protein	Embryo	362	-
Os02g54140.1	HSP20	18.6 kDa class III heat shock protein	Embryo	49	-
Os03g14180.1	HSP20	Chloroplastic 26.7 kDa heat shock protein	Common	-	−7.5
Os03g15960.1	HSP20	17.9 kDa class I heat shock protein	Common	-	−7.5
Os03g16020.1	HSP20	17.4 kDa class I heat shock protein	Endosperm	75	-
Os03g16030.1	HSP20	18.1 kDa class I heat shock protein	Embryo	394	-
Os03g16040.1	HSP20	17.7 kDa class I heat shock protein	Embryo	195	-
Os04g36750.1	HSP20	23.2 kDa heat shock protein	Embryo	93	-
Os06g11610.1	HSP20	Mitochondrial 26.2 kDa heat shock protein	Embryo	1,210	-
Os11g13980.1	HSP20	21.9 kDa heat shock protein	Embryo	1,350	-
Os01g08560.1	HSP70	70 kDa Heat shock protein	Common	-	−4.5
Os01g62290.1	HSP70	70 kDa Heat shock protein	Common	-	−3.6
Os02g48110.1	HSP70	70 kDa Heat shock protein	Common	-	−2.5
Os02g53420.1	HSP70	70 kDa Heat shock protein	Common	-	−2.1
Os03g02260.1	HSP70	70 kDa Heat shock protein	Embryo	135	-
Os03g11910.1	HSP70	70 kDa Heat shock protein	Embryo	218	-
Os03g16860.1	HSP70	70 kDa Heat shock protein	Embryo	280	-
Os03g16920.1	HSP70	70 kDa Heat shock protein	Common	-	−3.5
Os03g60620.1	HSP70	70 kDa Heat shock protein	Common	-	0.6
Os05g08840.1	HSP70	70 kDa Heat shock protein	Common	-	−4.0
Os05g23740.1	HSP70	70 kDa Heat shock protein	Common	-	0.8
Os05g38530.1	HSP70	70 kDa Heat shock protein	Embryo	206	-
Os11g47760.1	HSP70	70 kDa Heat shock protein	Common	-	−2.3
Os12g14070.1	HSP70	70 kDa Heat shock protein	Common	-	7.9
Os04g01740.1	HSP90	90kDa heat shock protein	Embryo	92	-
Os06g50300.1	HSP90	Endoplasmin homolog	Common	-	−2.9
Os08g38086.3	HSP90	90 kDa heat shock protein	Endosperm	109	-
Os08g39140.1	HSP90	Heat shock protein 81-1	Embryo	129	-
Os09g29840.1	HSP90	90 kDa heat shock protein	Common	-	0.5
Os09g30412.1	HSP90	Heat shock protein 81-2	Embryo	551	-
Os09g30418.1	HSP90	Heat shock protein 81-3	Common	-	0.3
Os12g32986.1	HSP90	90 kDa heat shock protein	Embryo	179	-
Os01g06630.1	LEA	Late embryogenesis abundant LEA_5	Embryo	21	-
Os01g12580.1	LEA	Late embryogenesis abundant LEA_2	Common	-	−5.5
Os01g50700.1	LEA	Dehydrin Rab25	Common	-	−4.4
Os01g50910.1	LEA	Late embryogenesis abundant LEA_4	Common	-	−6.9
Os02g15250.1	LEA	Late embryogenesis abundant protein	Common	-	−4.5
Os02g44870.1	LEA	Dehydrin	Embryo	723	-
Os03g06360.1	LEA	Late embryogenesis abundant protein	Common	-	−7.2
Os03g20680.1	LEA	Late embryogenesis abundant protein 1	Common	-	−4.7
Os03g53620.1	LEA	Late embryogenesis abundant protein	Embryo	262	-
Os03g62620.2	LEA	Late embryogenesis abundant LEA_2	Common	-	−3.3
Os04g52110.1	LEA	Late embryogenesis abundant protein	Common	-	−5.0
Os05g28210.1	LEA	Embryonic abundant protein 1	Embryo	14	-
Os05g46480.1	LEA	Late embryogenesis abundant protein	Common	-	−5.9
Os05g50710.1	LEA	Late embryogenesis abundant LEA_2	Embryo	321	-
Os06g23350.1	LEA	Late embryogenesis abundant protein	Common	-	−4.5
Os08g23870.1	LEA	Late embryogenesis abundant LEA_1	Embryo	110	-
Os11g26570.1	LEA	Dehydrin	Common	-	−2.98
Os11g26750.1	LEA	Dehydrin Rab16D	Embryo	1,396	-
Os11g26780.1	LEA	Dehydrin Rab16B GN = RAB16B	Embryo	330	-
Os12g43140.1	LEA	Late embryogenesis abundant protein	Embryo	69	-
Os02g43020.1	TPR	Heat shock protein stress-induced TPLR_2	Common	-	−2.4
Os08g34150.1	Lipocalin	OsTIL-2 Temperature-induced lipocalin 2	Embryo	644	-
Os02g39930.1	Lipocalin	OsTIL-1 Temperature-induced lipocalin 1	Embryo	1,424	-
Os03g31300.1	clpA/clpB	Chloroplastic chaperone protein ClpB2	Common	-	−0.12
Os02g08490.1	clpA/clpB	Mitochondrial chaperone protein ClpB3	Embryo	165	-

a *Rank of the protein respectively to the other tissue-specific proteins*.

b *The average seed protein abundance in each tissue (endosperm or embryo) was used to calculate the log_2_ ratio. A positive log_2_ ratio indicates an endosperm-favored metabolite accumulation (negative for embryo-favored log_2_ ratio)*.

#### Protein repair systems

Several enzymes involved in protein repair, were presently detected specifically in the rice embryo proteome. This was the case for three Methionine Sulfoxide Reductases (MSR) proteins namely MSRB5, MSRA2-1 and MSRA4 (Table S3C; Rouhier et al., 2006). MSRs are involved in the reversal of oxidized Met residues (Met sulfoxide, MetSO) in altered proteins thereby preventing aging-associated diseases in all organisms (Moskovitz, 2005). The MSRA4.1 is a plastidial enzyme potentially involved in oxidative stress resistance and that can repair free and protein-bound MetSO *in vitro* (Guo et al., 2009). MSR repair system in Medicago and Arabidopsis promote seed longevity (Châtelain et al., 2013). Secondly, the protein-L-isoaspartate *O*-methyltransferase can repair abnormal isoaspartyl occurring in damaged proteins (Thapar et al., 2001). In seeds, PIMT are actively involved with the maintenance of seed viability in Arabidopsis (Ogé et al., 2008) and rice (Petla et al., 2016). In wheat, PIMT activity is very high in dry mature seeds, increase up to 4 h after imbibition and then decrease during subsequent germination (Mudgett and Clarke, 1994). Amongst the two rice *PIMT* genes, we found the OsPIMT2 (Os04g40540; Petla et al., 2016) among the most abundant embryo-specific proteins (rank #188). More precisely, this could be ΔOsPIMT2, a truncated yet functional version of OsPIMT2 (Petla et al., 2016). This rice PIMT protein is accumulated during the very late stages of seed development in relation due to the formation of aspartyl residues during desiccation (Petla et al., 2016).

#### ROS homeostasis

The control of Reactive Oxygen Species (ROS, e.g., H_2_O_2_) homeostasis during both desiccation and early germination is of paramount importance for seed vigor and longevity (Sattler et al., 2004; Bailly et al., 2008). Proteomic and Metabolomic results emphasized several mechanisms that could help the embryo to cope with desiccation-induced oxidative stress.

First, tocopherols and tocotrienols participate to seed longevity by limiting lipid peroxidation (Sattler et al., 2004). In our data, α- and γ-tocopherols were found to be preferentially, if not exclusively, accumulated in the embryo (Table 1). Several proteins involved in tocopherol biosynthesis pathway were specifically found in the embryo such as the 4-hydroxyphenylpyruvate dioxygenase (HPPD, Os02g07160; Table S3C), which is involved in the production of both plastoquinone and tocopherol essential for plant survival (Sano et al., 2016). In the same way, OsVTE1 protein (Os02g17650), that is responsible for γ-tocopherol synthesis, was specifically detected in the rice embryo (Table S3C). Finally, part of the same pathway, we found expression of Arabidopsis *VTE2* (Os06g44840) and *VTE3* (Os12g42090) homologs with a favored embryo gene expression (5 and 2.5 fold respectively, Table S2F).

Along with vitamin E, ascorbate is also a very important antioxidant molecule. Ascorbate (AsA) and dehydroascorbate (DHA) were specifically or favorably detected in the endosperm (Table 1 and Table S1). Ascorbate and DHA can be degraded to threonate upon non-enzymatic reaction with H_2_O_2_ or enzymatically. Interestingly, threonate is also present in high amounts in the endosperm compared to the embryo (Table 1). This suggests that a complete AsA to threonate pathway exist in the endosperm. In developing wheat kernels, ascorbate level decrease from mid- to final seed developmental stage and the ascorbate pool becomes progressively oxidized (Paradiso et al., 2012). From our data, it seems that ascorbate *de novo* synthesis could be restricted to the embryo since a putative mannose-1-phosphate guanyltransferase (Os03g11050) and the two GDP-mannose 3,5-epimerase 1 and 2 (GME1, GME2) are specifically detected in the embryo at similar abundances (Table S3C). In contrast, the ascorbate salvage pathway from monodehydroascorbate (MDHA) by MDHA reductase (MDHAR) is present in both rice endosperm and embryo (Table S3C). In addition, the conversion of DHA back to AsA is also possible thanks to DHA reductases (OsDHAR1, Os05g02530) present in both rice embryo and endosperm proteomes at similar levels (Table S3C). Ascorbate could interact with ABA metabolism and/or signaling to modulate seed germination ability. Indeed, exogenous application of low concentrations of ascorbate is able to rescue rice seed germination from abscisic acid treatment (Ye et al., 2012).

The present proteome reveals a wide diversity of antioxidant enzymes that are already present in the dry seed with an embryo-favored accumulation (Figure 8). These enzymes include 4 superoxide dismutases (SODCC1, SODCC2, SODA, SODCP, Table S3C), two embryo-specific catalases (CATA and CATB, Table S3C) and 11 embryo-specific peroxidases including several ascorbate peroxidase i.e., the cytosolic OsAPX2, the peroxisomal OsAPX4 and the stromal OsAPX7 (Table S3C). These results seem to argue in favor of a more abundant ROS detoxification enzymatic apparatus in the rice embryo.

### The mature seed is the crossroad of post-transcriptional and translational regulations essential for germination success

Studies in various species demonstrated that the developmental transition from a maturing to a germinating seed is the place of strong post-transcriptional and translational regulations (Gallardo et al., 2007; Hajduch et al., 2010; Verdier et al., 2013; Galland et al., 2014b; Layat et al., 2014). In this study, we investigated that post-transcriptional and translational regulations occuring in both tissues of the rice seed at the end of its development (Figure 5). Since germination *sensu stricto* in both monocot and dicot seeds is only dependent on mRNA translation (Rajjou et al., 2004; Sano et al., 2012), we took a closer look at the translational machinery at the tissue-level.

#### Stored mRNAs and the translational machinery

We were wondering whether these comparable translational activities in the endosperm and embryo relied on different translational machinery sets. Indeed, we could show that, in Arabidopsis germinating seeds, stored mRNAs were differentially translated (Galland et al., 2014b) making selective mRNA translation a way to distinguish stored and neosynthesized mRNAs. For these reasons, proteins involved in translation and present in the embryo and endosperm were screened. Thus, 292 proteins related to mRNA translation processes (BIN 2.2.1–2.2.4) were identified in the embryo (Table S3F, Figure 6D). Specifically, 109 different ribosomal proteins could be identified. Initiation of translation is controlled by specific cap-dependent initiation factors (Roy and von Arnim, 2013). First, the 43S pre-initiation complex is formed through association between the 40S ribosomal subunit, a charged methionyl-tRNA and the eIF1, eIF2, eIF3 and eIF5 translation initiation factors. A complete set of the 43S pre-initiation complex was retrieved in the proteomic data (Table S3F). Out of the eight eIF3 protein subunits (B-C-D-E-F-H-K-H) monitored, the present data showed that two isoforms of eIF3E (Os07g12110 and Os07g07250) and one isoform of eIF3F (Os05g01450) are restricted to the embryo. In Arabidopsis, the eIF3F is a key regulator of embryo development particularly in actively developing tissues (Xia et al., 2010). In addition, it interacts with eIF3E suggesting that the observed rice eIF3E/3F proteins would play significant roles during embryonic or post-embryonic cellular processes. Upon formation, the multifactor complex (MFC) associates with the 40S ribosome thereby establishing the 43S pre-initiation complex (Figure 6D).

In parallel, the mRNA cap is recognized by the eIF4F complex composed of both a eIF4E and a eIF4G protein family (Roy and von Arnim, 2013). In plants, a very important feature is the presence of isoforms of eIF4E and eIF4G named eIF(iso)4E and eIF(iso)4G. These different isoforms participate to the mRNA translational selectivity (Mayberry et al., 2009; Martinez-Silva et al., 2012). In our data, we found that the eIF4E and eIF4(iso)4E subunits, responsible for recognition of the mRNA cap, were restricted to the rice embryo and present in relatively similar abundances (Table S3F, Figure 6D). The 43S pre-initiation complex subsequently associates to the eIF4 complex and binds to the mRNA. Translation initiation factors eIF4A with ATP-dependent helicase activity unwind mRNA 5′UTR secondary structures. The eIF4A-1 and eIF4A-3 were detected in our proteomic data (Table S3F, Figure 6D). The eIF4A-1 translation initiation factor is present in both endosperm and embryo, but, in contrast to almost all translation initiation factors, this protein is significantly and strongly up-accumulated in the endosperm (Table S3F, Figure 6D). The eIF4A-3 protein was strictly observed in the embryo suggesting that embryo and endosperm use different eIF4A helicases during mRNA translation initiation. The translation initiation factor eIF6-2 interacts with RACK1, a negative regulator of ABA response and positive regulator of GA signaling (Guo et al., 2011; Fennell et al., 2012). Especially relevant in seed biology, it was demonstrated that ABA inhibited RACK1 and eIF6 gene expressions (Guo et al., 2011). In Arabidopsis, three homologs of the mammalian RACK1, namely RACK1A, RACK1B and RACK1C were characterized. In our proteomic description of rice seeds, it was interesting to observe the presence of two RACK1 proteins with OsRACK1A detected in both tissues and OsRACK1B only detected in the embryo along with the embryo-specific eIF6-2 (Table S3C, Figure 6D). Recently, the *OsRACK1A* gene has been shown to positively regulate rice seed germination through promotion of ABA catabolism and H_2_O_2_ synthesis (Zhang et al., 2014). Thus, in addition to RACK1A, RACK1B could also play a major role in the embryo during seed germination. RACK1A and B could also link ABA and GA signaling with mRNA translation. We only detected the eIF6-2 protein in the embryo suggesting that RACK1A/B regulations of mRNA translation only apply to the embryo and not to the endosperm. Together with the absence of the two cap recognition eIF4E proteins in the endosperm, it further confirms that embryo and endosperm may have contrasted qualitative mRNA translation regulations. Very recent evidences showed that eIFiso4G1 translation initiation factor has a role in the fatty acid profile of Arabidopsis developing seeds through the balance of plastic and nucleus-encoded mRNAs involved in fatty acid biosynthesis (Li et al., 2017). Moreover, this role of eIFisoG1 is not be compensated by eIFiso4G2 suggesting a very specific effect. Further work should distinguish the translational machinery at the tissue level and its consequences on seed metabolism.

## Concluding remarks

Starch and SSPs were long associated with the endosperm storage function. Thus, it was remarkable to pinpoint, in our proteomic dataset, the presence of glutelins and starch biosynthesis enzymes at non-negligible level also in the embryo (Figures 4, 7). These results refine and expand previous proteomic results on whole developing rice seeds (Koller et al., 2002). Altogether, this would also support the supernumerary embryo hypothesis with both ancestral tissues being equipped with different molecular apparatus before divergence. Classically, the inner SE has been seen as a dead storage tissue since the central parts of the endosperm undergo PCD (Young and Gallie, 2000). Yet, we could show that, as expected, the embryoless endosperm (aleurone and SE) showed an important translational activity however, the embryo display a higher protein synthesis (Figures 6B,C). While the functional consequences of this mRNA translational activity remain to be established, it is clear that the seed endosperm has emerging new roles regarding the control of seed germination and environmental adaptation (Yan et al., 2014; Bassel, 2016). New determinants of agricultural seed quality both in monocots and dicots crops will undoubtedly benefit from tissue-specific combined “multi-omics.” The genetic and tissue heterogeneity of the mature seed is a considerable challenge to seed biologists. In addition, the seed definitely constitute a fascinating plant organ in which post-transcriptional regulations and translational selectivity fine-tune the biological processes that are spatially and temporally regulated within a few hours. A renewed vision of seed biology by integrative systems biology would certainly dig out meaningful new genetic determinants of seed quality.

## Author contributions

MG, DH, IL, and LR designed and performed the experimental work. GCl completed the metabolome analyses. SB and SH realized the transcriptomic analysis while BV gathered the proteomic data. GC, BC were involved in the preparation of samples. FG, JT, and EA helped with R statistics. HM and BG provided help with cytological observations. MG and LR wrote the manuscript. All authors read and approved the final manuscript.

### Conflict of interest statement

The authors declare that the research was conducted in the absence of any commercial or financial relationships that could be construed as a potential conflict of interest. The reviewer ME and handling Editor declared their shared affiliation.

## Supplementary material

Figure S1 Protein SDS-PAGE gels used for label-free shotgun proteomics. **(A,B)** Proteins were extracted, quantified by the Bradford assay and 25 μg of embryo **(A)** or endosperm **(B)** proteins were subjected to SDS-PAGE (10% acrylamide) and Coomassie staining. **(C)** Protein quantification in each lane according to the Multi Gauge 3.0 software. Areas quantified are shown on the gels.

Figure S2 Transcriptome **(A)** and proteome **(B)** analysis workflows applied in this study.

Figure S3 Gene Singular Enrichment Analysis of genes preferentially expressed in the endosperm. From the list of the differentially expressed genes, we extracted 787 probe sets with a preferred expression in the endosperm (log_2_ ratio > +1.4, *p* < 0.01). The resulting list of 787 probe sets was then submitted to the AgriGO Gene Singular Enrichment Analysis (Hypergeometric test corrected by Yekutieli False Discovery Rate correction, Affymetrix Rice Genome Array set as background, *p* < 0.05) to detect enriched Biological Process **(A)**, Cellular Compartment **(B)**, and Molecular Function **(C)** GO categories. 722 probes were classified. Light yellow, dark yellow and red boxes indicates GO terms significant enrichment at *p* < 0.05, < 0.01, and < 0.001 respectively.

Figure S4 Gene Singular Enrichment Analysis of genes preferentially expressed in the embryo. From the list of the differentially expressed genes, we extracted 1,921 probe sets with a preferred expression in the embryo (log_2_ ratio< −1, *p* < 0.01). The resulting list of 1,921 probe sets was then submitted to the AgriGO Gene Singular Enrichment Analysis (Hypergeometric test corrected by Yekutieli False Discovery Rate correction, Affymetrix Rice Genome Array set as background, *p* < 0.05) to detect enriched Biological Process **(A)**, Cellular Compartment **(B)**, and Molecular Function **(C)** GO categories. 1,712 probes were classified. Light yellow, dark yellow and red boxes indicates GO terms significant enrichment at *p* < 0.05, < 0.01, and < 0.001 respectively.

Figure S5 Embryo and endosperm peptide and protein distributions. **(A–D)** Distributions of the gene-specific peptide and protein abundances from **(A,C)** embryo and **(B,D)** endosperm on a log_2_-transformed axis. **(E,F)** Quantile-quantile comparison of embryo and endosperm **(E)** peptide and **(F)** protein abundances (log_2_-transformed). **(G)** Distribution of the log_2_ ratios (endosperm vs. embryo) for the 673 common proteins. The comparison with the theoretical normal law (red line with a mean equal to the estimated mean i.e., −1.89 and standard deviation 2.89) is shown.

Figure S6 Gene Singular Enrichment Analysis of endosperm-specific or endosperm-favored proteins. The list of the 113 endosperm-specific and 76 endosperm-favored (log_2_ ratio > 1.7) was subjected to a Gene Singular Enrichment Analysis tool (hypergeometric test corrected by Yekutieli False Discovery rate, whole transcriptome set as background, *p* < 0.05) to detect enriched endosperm protein Biological Process **(A)**, Cellular Compartment **(B)**, and Molecular Function **(C)** GO categories. 164 proteins were classified. Light yellow, dark yellow and red boxes indicates GO terms significant enrichment at *p* < 0.05, < 0.01, and < 0.001 respectively.

Figure S7 Gene Singular Enrichment Analysis of embryo-specific or embryo-favored proteins. The list of the 1,426 embryo-specific and 267 embryo-favored (log_2_ ratio < −2.9) was subjected to a Gene Singular Enrichment Analysis tool (hypergeometric test corrected by Yekutieli False Discovery rate, whole transcriptome set as background, *p* < 0.05) to detect enriched embryo protein Biological Process **(A)**, Cellular Compartment **(B)**, and Molecular Function **(C)** GO categories. 1,627 proteins were classified. Light yellow, dark yellow and red boxes indicates GO terms significant enrichment at *p* < 0.05, < 0.01, and < 0.001 respectively.

## Abbreviations

AL: aleurone layer
ALDH: aldehyde dehydrogenase
AsA: ascorbate
DHA: dehydroascorbate
DT: desiccation tolerance
eIF: eukaryotic initiation factor
ESR: embryo surrounding region
ETC: endosperm transfer cells
HAI: hours after imbibition
HSP: heat shock protein
LEA: late embryogenesis abundant
LOX: lipoxygenase
MDA: malondialdehyde
MDHA: monodehydroascorbate
MSR: methionine sulfoxide oxidase
NBB: naphthol blue black
PA: periodic acid
PCD: programmed cell death
PIMT: protein-L-isoaspartate *O*-methyltransferase
PLD: phospholipase D
QTL: quantitative trait locus
RFO: raffinose family oligosaccharides
ROS: reactive oxygen species
SE: starchy endosperm
SSPs: seed storage proteins
TAGs: triacylglycerols
TCA: tricarboxylic acid cycle
TIC: total ion current
XIC: extracted ion current.
