# Supplementary figures and images for: An Integrated “Multi-Omics” Comparison of Embryo and Endosperm Tissue-Specific Features and Their Impact on Rice Seed Quality
Source: Front Plant Sci. 2017 Nov 22;8:1984. doi: 10.3389/fpls.2017.01984 (PMC5702907; doi:10.3389/fpls.2017.01984)

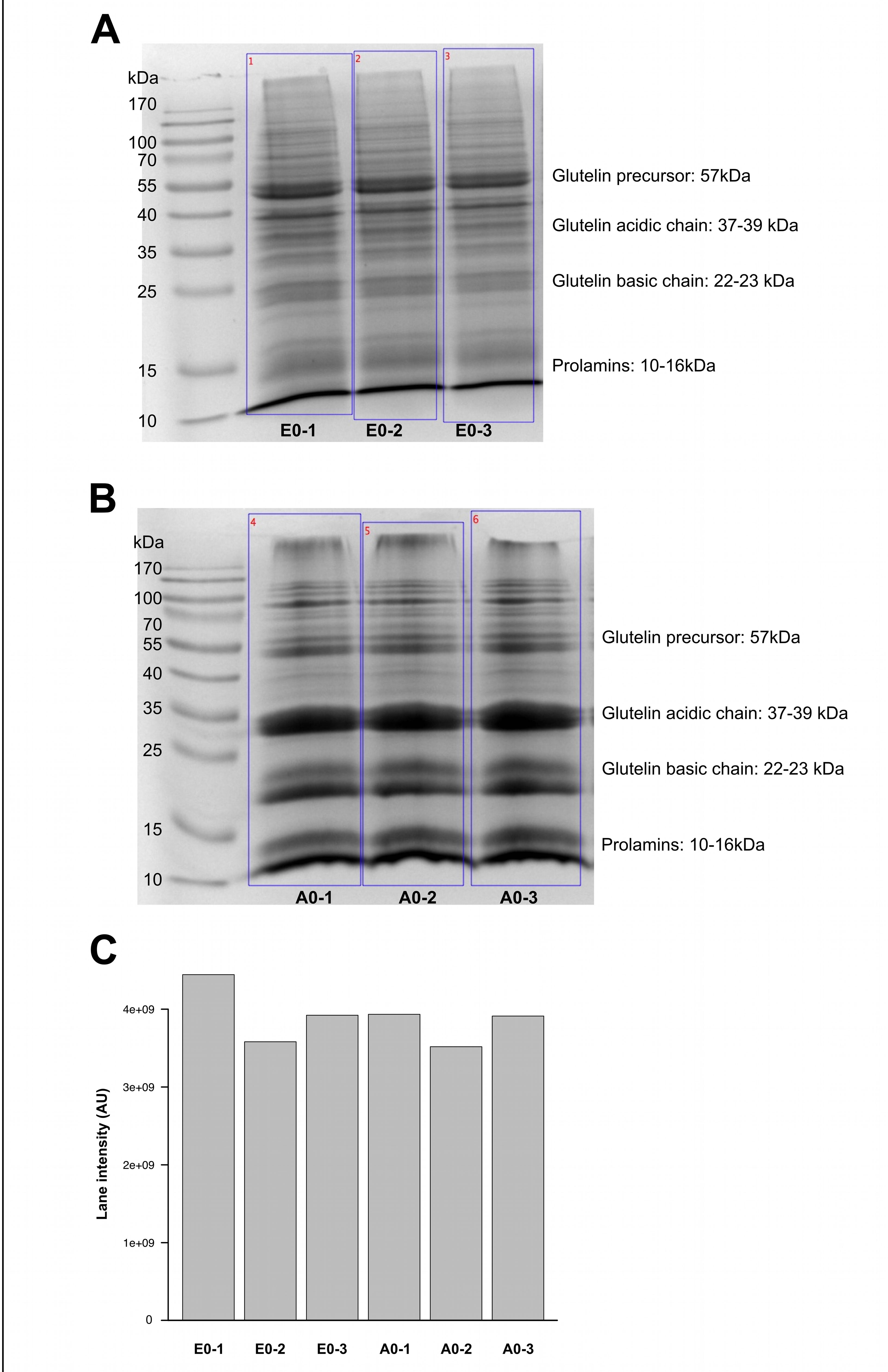

Supplement: Figure S1 — Protein SDS-PAGE gels used for label-free shotgun proteomics. (A,B) Proteins were extracted, quantified by the Bradford assay and 25 μg of embryo (A) or endosperm (B) proteins were subjected to SDS-PAGE (10% acrylamide) and Coomassie staining. (C) Protein quantification in each lane according to the Multi Gauge 3.0 software. Areas quantified are shown on the gels. [file Image1.JPEG]

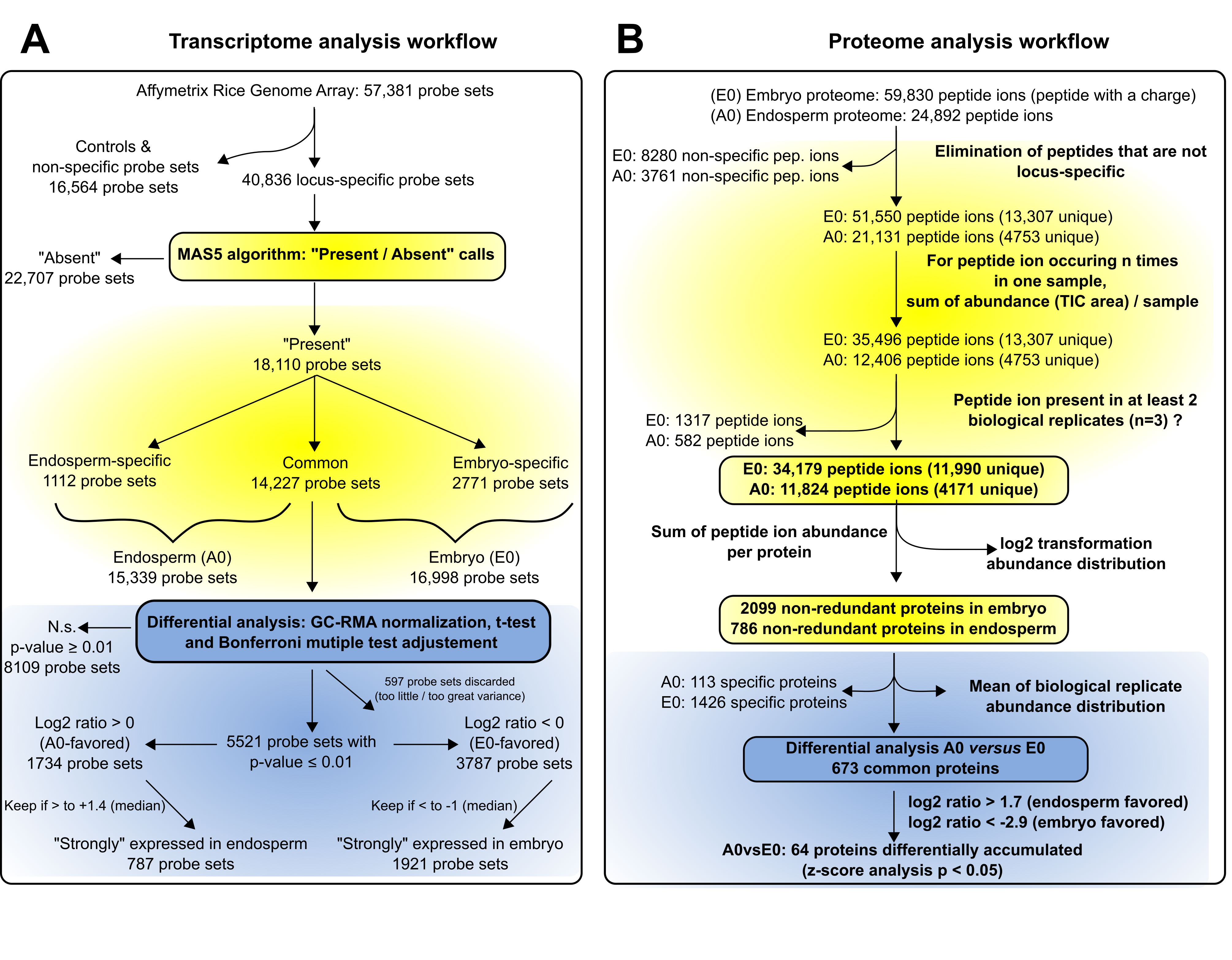

Supplement: Figure S2 — Transcriptome (A) and proteome (B) analysis workflows applied in this study. [file Image2.JPEG]

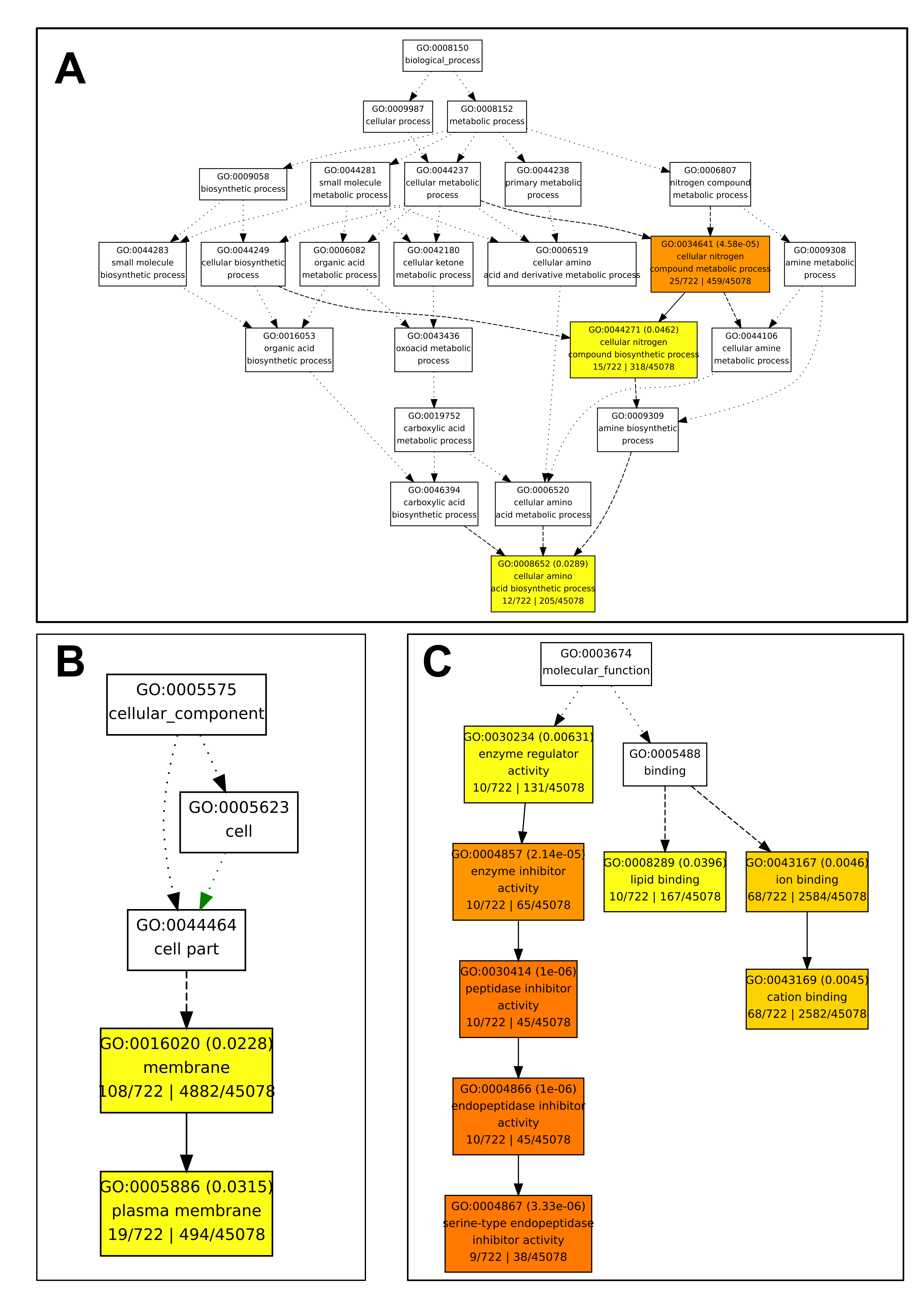

Supplement: Figure S3 — Gene Singular Enrichment Analysis of genes preferentially expressed in the endosperm. From the list of the differentially expressed genes, we extracted 787 probe sets with a preferred expression in the endosperm (log2 ratio > +1.4, p < 0.01). The resulting list of 787 probe sets was then submitted to the AgriGO Gene Singular Enrichment Analysis (Hypergeometric test corrected by Yekutieli False Discovery Rate correction, Affymetrix Rice Genome Array set as background, p < 0.05) to detect enriched Biological Process (A), Cellular Compartment (B), and Molecular Function (C) GO categories. 722 probes were classified. Light yellow, dark yellow and red boxes indicates GO terms significant enrichment at p < 0.05, < 0.01, and < 0.001 respectively. [file Image3.JPEG]

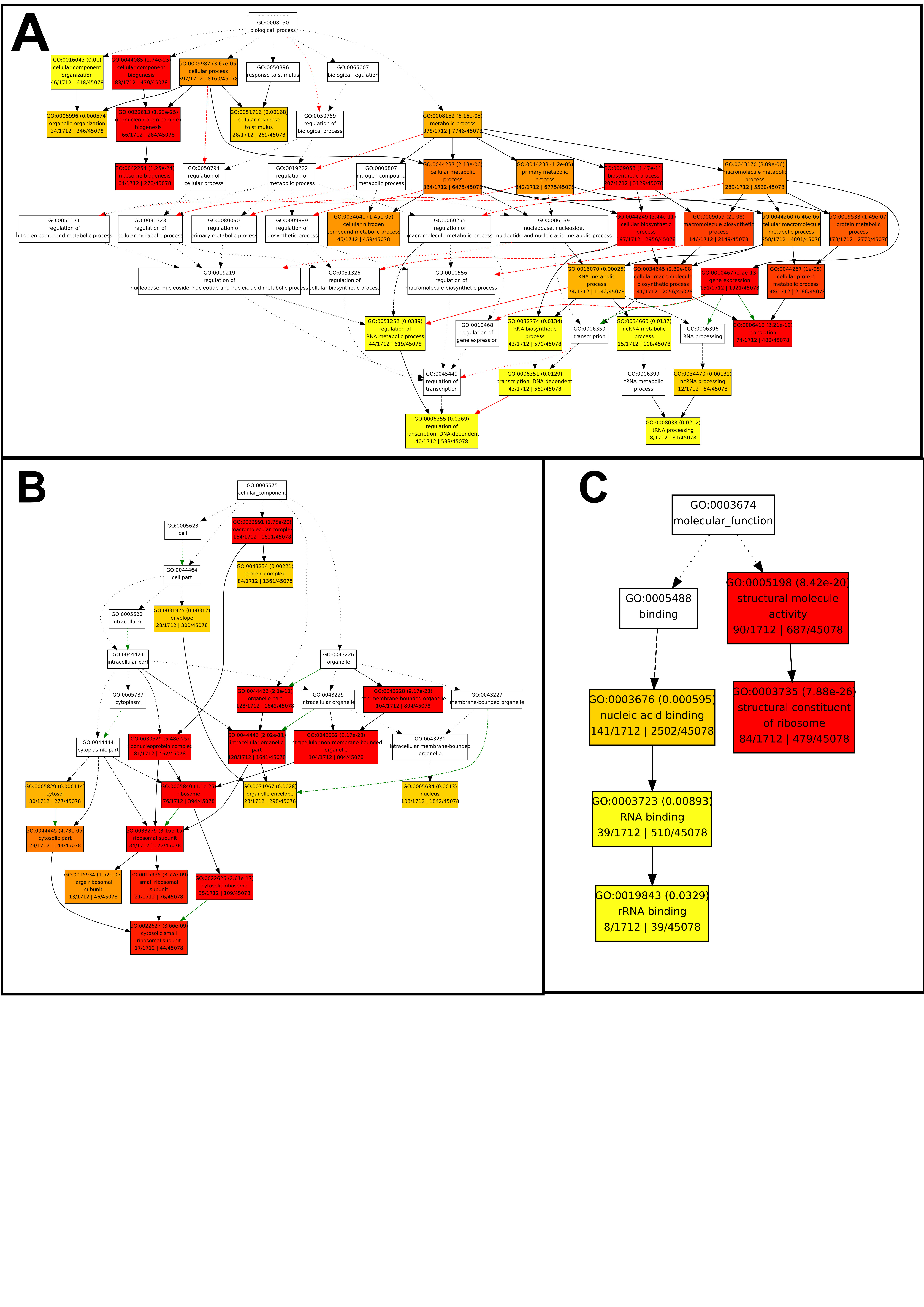

Supplement: Figure S4 — Gene Singular Enrichment Analysis of genes preferentially expressed in the embryo. From the list of the differentially expressed genes, we extracted 1,921 probe sets with a preferred expression in the embryo (log2 ratio< −1, p < 0.01). The resulting list of 1,921 probe sets was then submitted to the AgriGO Gene Singular Enrichment Analysis (Hypergeometric test corrected by Yekutieli False Discovery Rate correction, Affymetrix Rice Genome Array set as background, p < 0.05) to detect enriched Biological Process (A), Cellular Compartment (B), and Molecular Function (C) GO categories. 1,712 probes were classified. Light yellow, dark yellow and red boxes indicates GO terms significant enrichment at p < 0.05, < 0.01, and < 0.001 respectively. [file Image4.JPEG]

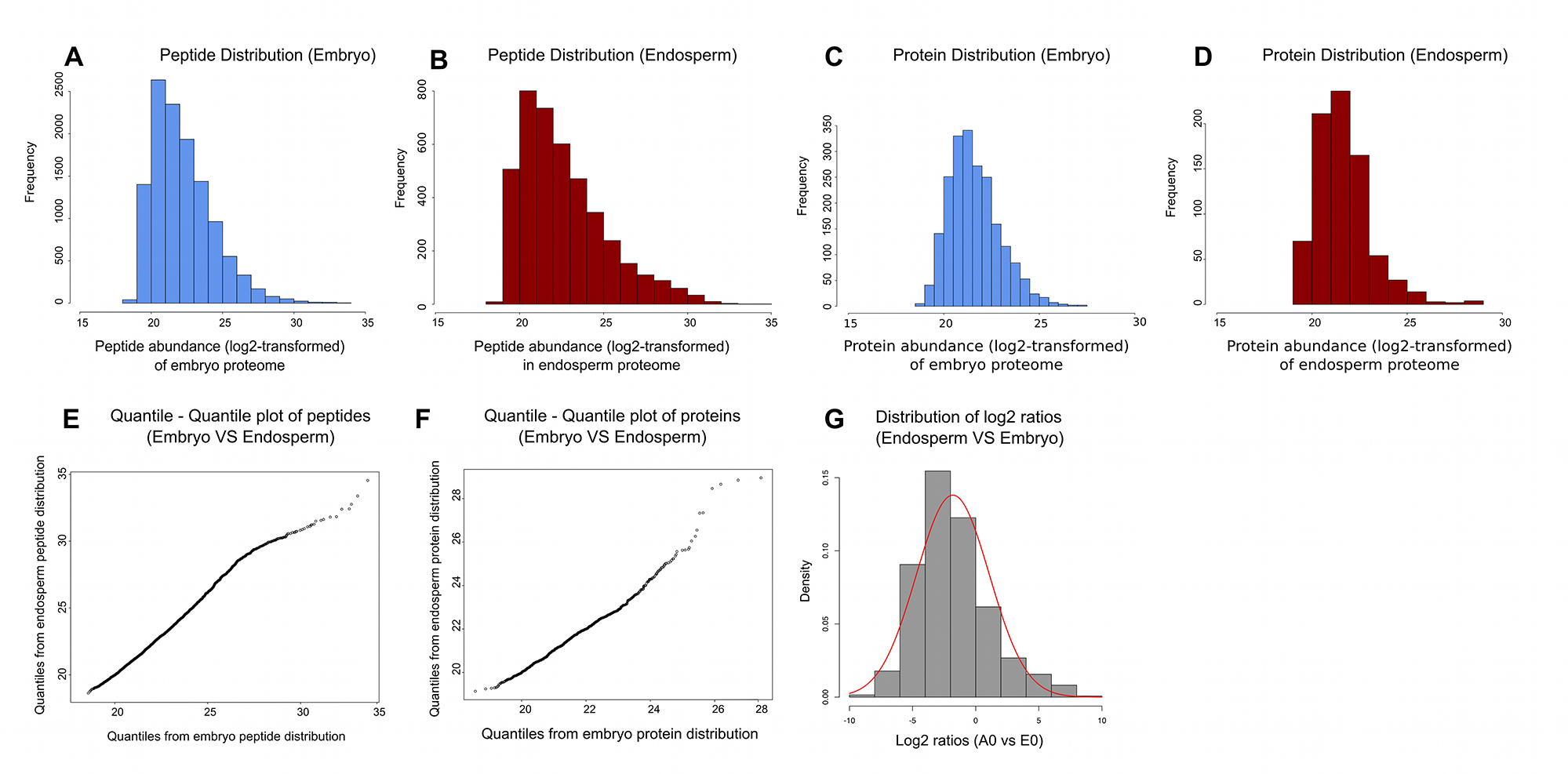

Supplement: Figure S5 — Embryo and endosperm peptide and protein distributions. (A–D) Distributions of the gene-specific peptide and protein abundances from (A,C) embryo and (B,D) endosperm on a log2-transformed axis. (E,F) Quantile-quantile comparison of embryo and endosperm (E) peptide and (F) protein abundances (log2-transformed). (G) Distribution of the log2 ratios (endosperm vs. embryo) for the 673 common proteins. The comparison with the theoretical normal law (red line with a mean equal to the estimated mean i.e., −1.89 and standard deviation 2.89) is shown. [file Image5.TIF]

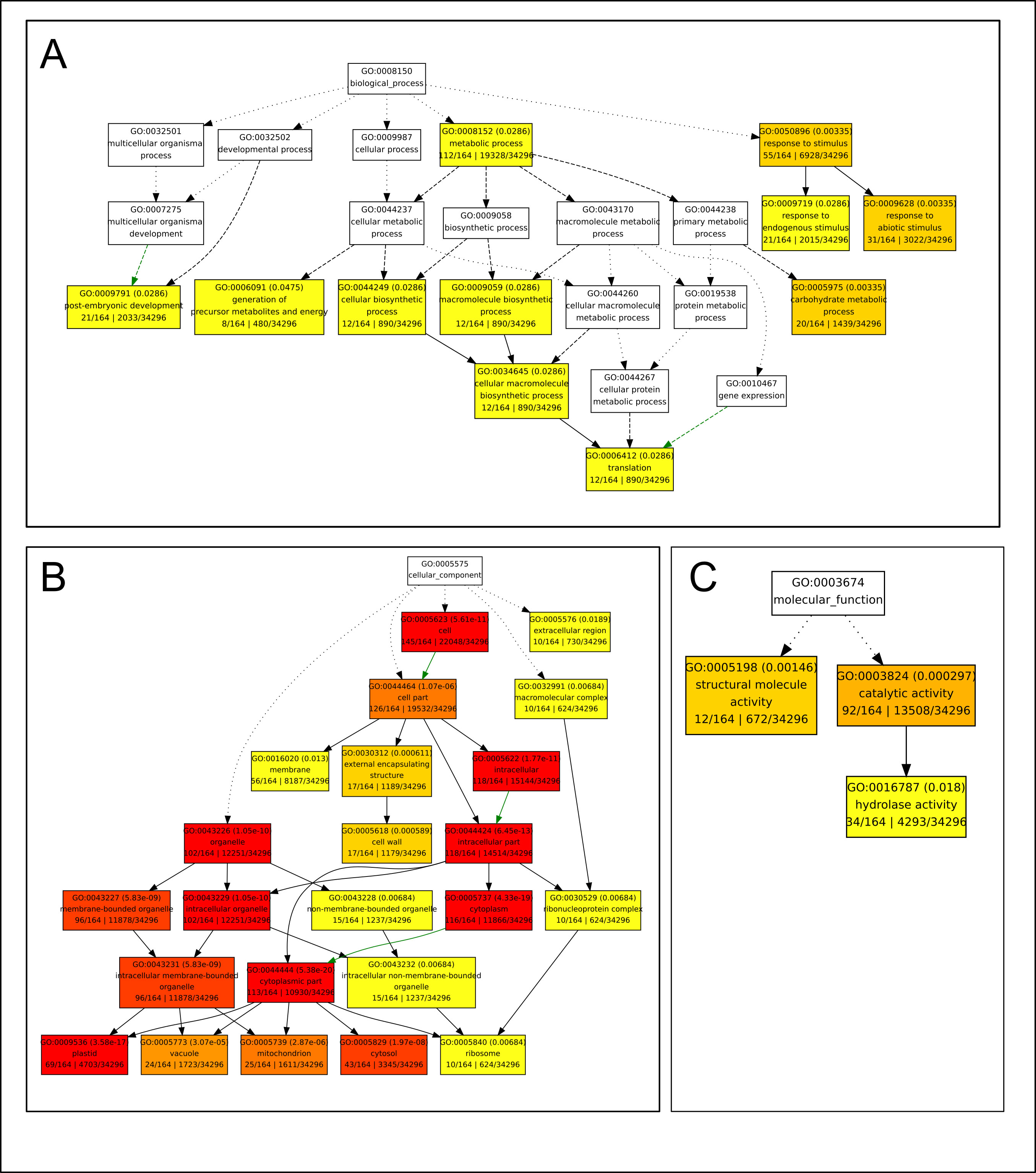

Supplement: Figure S6 — Gene Singular Enrichment Analysis of endosperm-specific or endosperm-favored proteins. The list of the 113 endosperm-specific and 76 endosperm-favored (log2 ratio > 1.7) was subjected to a Gene Singular Enrichment Analysis tool (hypergeometric test corrected by Yekutieli False Discovery rate, whole transcriptome set as background, p < 0.05) to detect enriched endosperm protein Biological Process (A), Cellular Compartment (B), and Molecular Function (C) GO categories. 164 proteins were classified. Light yellow, dark yellow and red boxes indicates GO terms significant enrichment at p < 0.05, < 0.01, and < 0.001 respectively. [file Image6.JPEG]

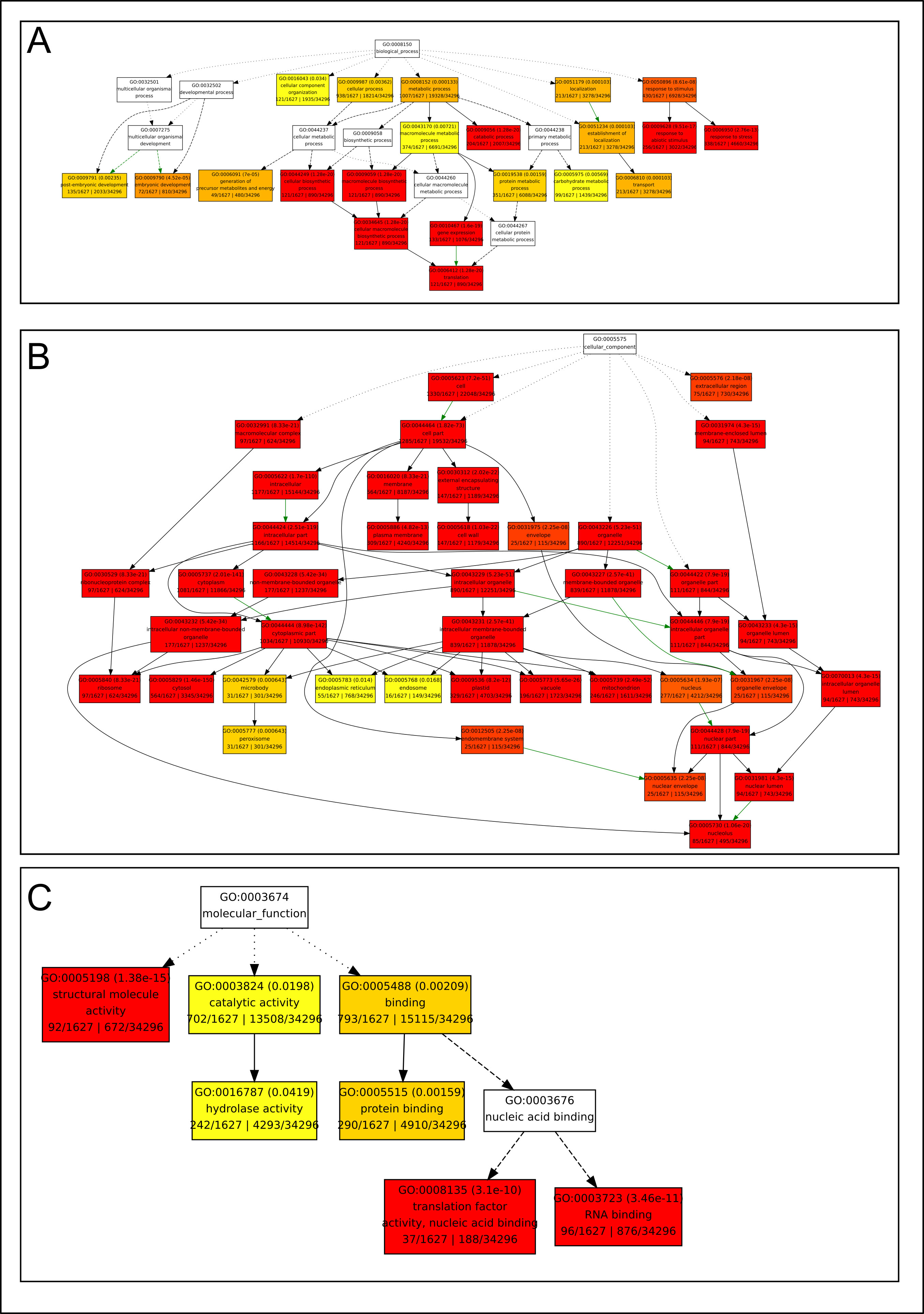

Supplement: Figure S7 — Gene Singular Enrichment Analysis of embryo-specific or embryo-favored proteins. The list of the 1,426 embryo-specific and 267 embryo-favored (log2 ratio < −2.9) was subjected to a Gene Singular Enrichment Analysis tool (hypergeometric test corrected by Yekutieli False Discovery rate, whole transcriptome set as background, p < 0.05) to detect enriched embryo protein Biological Process (A), Cellular Compartment (B), and Molecular Function (C) GO categories. 1,627 proteins were classified. Light yellow, dark yellow and red boxes indicates GO terms significant enrichment at p < 0.05, < 0.01, and < 0.001 respectively. [file Image7.JPEG]
